# Efficacy of psychological and behavioral interventions for depressive symptoms in people with epilepsy: a systematic review and meta-analysis

**DOI:** 10.3389/fmed.2026.1917962

**Published:** 2026-09-17

**Authors:** Yiming Sun, Jie Wang, Yufu Guan, Zhangyi Wu

**Affiliations:** 1 Department of Neurosurgery, Tongde Hospital of Zhejiang Province, Hangzhou, Zhejiang, China; 2 Heart Emergency Ward, Affiliated Zhongshan Hospital of Dalian University, Dalian, Liaoning, China; 3 Department of Breast Surgery, Affiliated Zhongshan Hospital of Dalian University, Dalian, Liaoning, China

**Keywords:** cognitive behavioral therapy, depression, epilepsy, meta-analysis, mindfulness, progressive muscle relaxation, self-management

## Abstract

**Background:**

Depression is a common and clinically important comorbidity in people with epilepsy and is associated with poorer quality of life, impaired treatment adherence, increased disease burden, and worse clinical outcomes. Psychological and behavioral interventions have been increasingly applied in this population; however, the overall efficacy and consistency of evidence across different intervention categories remain uncertain.

**Objective:**

This systematic review and meta-analysis aimed to evaluate the effects of psychological and behavioral interventions on depressive symptoms in people with epilepsy, with particular attention to cognitive behavioral therapy, mindfulness-based interventions, self-management or collaborative-care programs, and relaxation-based interventions.

**Methods:**

A systematic literature search was conducted in PubMed, Embase, Web of Science, MEDLINE, and Cochrane Library for studies published between January 2005 and May 2026. Randomized controlled trials and controlled clinical studies evaluating eligible interventions for depression-related outcomes in people with epilepsy were included. The primary outcome was depression severity measured using validated rating scales. Standardized mean differences (SMDs) with 95% confidence intervals (CIs) were calculated for continuous outcomes. Random-effects models were used to account for clinical and methodological heterogeneity. Risk of bias and certainty of evidence were assessed using the Cochrane RoB 2 tool and the GRADE framework, respectively.

**Results:**

Twenty-two studies were included in the systematic review, of which 11 contributed data to the quantitative synthesis of continuous depression severity outcomes. Overall, active psychological and behavioral interventions were associated with significantly greater reductions in depressive symptoms compared with treatment as usual or control conditions (SMD = −0.63, 95% CI: −0.90 to −0.36). In subgroup analyses, cognitive behavioral therapy showed a significant beneficial effect (SMD = −0.55, 95% CI: −0.90 to −0.21), as did mindfulness-based interventions (SMD = −0.46, 95% CI: −0.86 to −0.07) and self-management or collaborative-care interventions (SMD = −0.43, 95% CI: −0.74 to −0.12). Progressive muscle relaxation showed a large effect in a single small study (SMD = −1.65), but this finding should be interpreted cautiously because of limited evidence. Most studies were judged as having some concerns regarding risk of bias, mainly due to lack of blinding, reliance on self-reported outcomes, and incomplete outcome reporting. The certainty of evidence ranged from low to very low across intervention categories.

**Conclusion:**

Psychological and behavioral interventions may help reduce depressive symptoms in people with epilepsy. Among the available intervention categories, cognitive behavioral therapy and self-management or collaborative-care interventions showed the most consistent and clinically interpretable evidence. Mindfulness-based interventions may also be beneficial, although the overall certainty of evidence remained low. Findings for progressive muscle relaxation remain preliminary. Further well-designed, adequately powered randomized trials with standardized outcome measures and longer follow-up are needed to confirm these findings.

**Systematic Review Registration:**

https://www.crd.york.ac.uk/PROSPERO/view/CRD420261403546, PROSPERO CRD420261403546.

## 1 Introduction

Epilepsy is a common chronic neurological disorder characterized by a persistent predisposition to generate epileptic seizures and by its associated neurobiological, cognitive, psychological, and social consequences (1). It affects approximately 50 million people worldwide and represents a substantial global health burden (1). In addition to recurrent seizures, people with epilepsy frequently experience psychiatric comorbidities, among which depression is one of the most common and clinically important (2, 3). The reported prevalence of depressive symptoms in people with epilepsy varies across populations and assessment methods, but depression is consistently recognized as a major contributor to impaired quality of life, reduced treatment adherence, worse seizure-related outcomes, increased healthcare utilization, and elevated suicide risk (4–7).

The relationship between epilepsy and depression is complex and bidirectional. Shared neurobiological mechanisms, including limbic system dysfunction, alterations in neurotransmitter pathways, inflammatory processes, and the effects of antiseizure medications, may contribute to the development of depressive symptoms in people with epilepsy (6, 8–11). Psychosocial factors, such as stigma, reduced social participation, unemployment, limited functional independence, and concerns about unpredictable seizures, may further increase vulnerability to depression (5, 7, 8). Because depression may aggravate disease burden and interfere with epilepsy management, effective identification and treatment of depressive symptoms should be considered an important component of comprehensive epilepsy care (12).

A variety of interventions have been investigated for depressive symptoms in people with epilepsy. These include cognitive behavioral therapy, internet-based psychological interventions, mindfulness-based programs, self-management and collaborative-care interventions, psychoeducation, progressive muscle relaxation, antidepressant pharmacotherapy, antiseizure medication-related strategies, and neuromodulation-based approaches such as transcranial direct current stimulation (13–34). However, the available evidence remains heterogeneous. Studies differ in population characteristics, intervention content, control conditions, follow-up duration, depression scales, and outcome definitions. In addition, many trials are small, and several intervention categories are supported by only a limited number of studies. These factors make it difficult for clinicians to determine which types of interventions have the most consistent evidence for improving depressive symptoms in people with epilepsy.

Previous systematic reviews and meta-analyses have evaluated selected psychological or behavioral interventions in people with epilepsy and suggested potential benefits of cognitive behavioral therapy, mindfulness-based interventions, and self-management approaches for improving depressive symptoms and psychological well-being (35–37). However, several important gaps remain. First, previous reviews have often focused on specific intervention categories, such as cognitive behavioral therapy or mindfulness-based interventions, while broader reviews have also examined multiple non-pharmacological approaches in people with epilepsy. However, important gaps remain in the depression-specific evidence base (35, 36). Second, some previous reviews have combined depression outcomes with broader psychosocial outcomes, including quality of life, anxiety, and coping-related measures, which may limit the interpretation of intervention effects specifically on depressive symptoms (36, 37). Third, clinically meaningful dichotomous depression-related outcomes, including clinically significant depressive symptoms, depression incidence, treatment response, and remission, have not been consistently synthesized together with continuous depression severity outcomes. Furthermore, the durability of treatment effects across different follow-up periods remains insufficiently explored, as previous studies have reported heterogeneous follow-up durations and inconsistent assessments of long-term treatment benefits (35, 37). Therefore, an updated systematic review focusing specifically on depression-related outcomes is needed to provide a comprehensive evaluation of the efficacy and consistency of psychological and behavioral interventions for depressive symptoms in people with epilepsy. Therefore, we conducted a systematic review and meta-analysis to comprehensively evaluate the effects of psychological and behavioral interventions on depressive symptoms in people with epilepsy. Unlike previous reviews that primarily focused on individual intervention categories or broader psychosocial outcomes, we synthesized evidence across clinically relevant intervention categories and separately evaluated continuous depression severity outcomes and dichotomous depression-related outcomes. Overall effects were estimated for active psychological and behavioral interventions compared with treatment as usual (TAU) or control conditions, and subgroup analyses were conducted according to intervention category to identify approaches with the most consistent evidence for reducing depressive symptoms.

## 2 Methods

### 2.1 Study design

This study was designed as a systematic review and pairwise meta-analysis to evaluate the efficacy of psychological and behavioral interventions for depressive symptoms in people with epilepsy. The primary interventions of interest included cognitive behavioral therapy, mindfulness-based interventions, self-management or collaborative-care programs, and relaxation-based interventions. Pharmacological, antiseizure medication-related, and neuromodulation-based interventions were considered eligible during the systematic review stage when they reported depression-related outcomes, but they were summarized separately when they were not clinically comparable with the primary psychological and behavioral intervention categories.

The review was conducted and reported in accordance with the Preferred Reporting Items for Systematic Reviews and Meta-Analyses 2020 statement. The study protocol was registered in the International Prospective Register of Systematic Reviews, PROSPERO, under the registration number CRD420261403546.

The primary outcome was depression severity assessed using validated depression rating scales. Secondary outcomes included dichotomous depression-related outcomes, including clinically significant depressive symptoms, depression incidence, treatment response, and remission.

### 2.2 Objectives

The primary objective was to evaluate the efficacy of eligible interventions in reducing depressive symptom severity among people with epilepsy. The main comparison of interest was active intervention versus treatment as usual, TAU, wait-list control, no intervention, placebo, sham control, or other relevant control conditions.

The secondary objectives were to summarize dichotomous depression-related outcomes, including clinically significant depressive symptoms, depression incidence, response, and remission; to explore whether intervention effects differed across intervention categories; to evaluate heterogeneity across studies; and to assess the certainty of evidence using the GRADE framework. Continuous and dichotomous outcomes were analyzed separately because they differed in effect measures and clinical interpretation.

### 2.3 PICOS framework

The eligibility criteria were structured according to the PICOS framework. Eligible participants were people with epilepsy, regardless of whether depressive symptoms or a depressive disorder were required as a baseline inclusion criterion. Studies were eligible if they reported at least one validated depression-related outcome, including continuous depression severity scores or dichotomous depression-related outcomes such as clinically significant depressive symptoms, depression incidence, treatment response, or remission.（ Table 1）Eligible interventions for the primary analysis included psychological and behavioral interventions, such as cognitive behavioral therapy, internet-based CBT, psychotherapy, psychoeducation, cognitive-behavioral intervention, mindfulness-based interventions, self-management or collaborative-care programs, and relaxation-based interventions. Specific interventions included, but were not limited to, UPLIFT, mindfulness-based intervention, mindful breathing, PEARLS, TIME, SMART, pharmaceutical care, multidisciplinary management, solution-focused therapy, and progressive muscle relaxation. Pharmacological interventions, antiseizure medication-related comparisons, and neuromodulation-based interventions, such as antidepressant pharmacotherapy, antiseizure medication comparisons, and transcranial direct current stimulation, were considered eligible for the systematic review when they reported depression-related outcomes, but were summarized separately when they were not clinically comparable with the primary psychological and behavioral intervention categories.

**Table 1:** PICOS framework for the systematic review and meta-analysis.

Element	Description
Population	People of any age with a clinical diagnosis of epilepsy, with or without baseline depressive symptoms, provided that depressive outcomes were assessed using validated instruments.
Intervention	Eligible interventions included psychological or behavioral interventions, such as CBT, internet-based CBT, psychotherapy, psychoeducation, and cognitive-behavioral intervention; mindfulness-based interventions, including UPLIFT, mindfulness-based intervention, and mindful breathing; self-management or collaborative-care interventions, including PEARLS, TIME, SMART, pharmaceutical care, multidisciplinary management, and solution-focused therapy; relaxation-based interventions such as progressive muscle relaxation; pharmacological interventions such as antidepressants or antiseizure medication-related comparisons; and neuromodulation-based interventions such as tDCS.
Comparator	Eligible comparators included treatment as usual, TAU, care as usual, standard antiepileptic treatment, wait-list control, no intervention, placebo control, sham control, and alternative active interventions. For quantitative synthesis, comparisons were pooled only when interventions and comparators were clinically comparable. Comparisons supported by a single study or involving heterogeneous active comparators were summarized narratively.
Outcomes	The primary outcome was depression severity assessed using validated depression rating scales, including but not limited to BDI, HAM-D, MADRS, PHQ-9, QIDS, NDDI-E, CES-D, and HSCL-20. Secondary outcomes included dichotomous depression-related outcomes, such as clinically significant depressive symptoms, depression incidence, response, and remission. Quality of life, anxiety, adherence, and seizure-related outcomes were extracted when available but were not the primary focus of the present meta-analysis.
Study design	Randomized controlled trials and controlled clinical intervention studies with extractable comparative outcome data were eligible. Studies had to report sufficient data for effect-size estimation, including mean and standard deviation, standard error, confidence interval, event counts, or risk estimates. Case reports, uncontrolled single-arm studies without extractable comparative data, reviews, editorials, protocols, conference abstracts without sufficient data, animal studies, and duplicate reports were excluded.

Eligible comparators included TAU, usual care/care as usual, standard antiepileptic treatment, wait-list control, no-intervention control, placebo control, sham control, or alternative active interventions. In individual studies, “usual care” and “care as usual” were considered equivalent terms referring to routine clinical management provided in the original study setting without the evaluated intervention. For quantitative synthesis, comparisons were pooled only when intervention categories and comparator conditions were clinically comparable. The primary outcome was depression severity assessed using validated depression rating scales, including but not limited to BDI, HAM-D, MADRS, PHQ-9, QIDS, NDDI-E, CES-D, and HSCL-20. Secondary outcomes included dichotomous depression-related outcomes, such as clinically significant depressive symptoms, depression incidence, treatment response, and remission. Quality of life, anxiety, treatment adherence, and seizure-related outcomes were extracted when available but were not the primary focus of the present meta-analysis.

Randomized controlled trials and controlled intervention studies with extractable comparative outcome data were eligible. Studies had to report sufficient data for effect-size estimation, including mean and standard deviation, standard error, confidence interval, change score, event counts, or risk estimates. Case reports, uncontrolled single-arm studies without extractable comparative data, reviews, editorials, protocols, conference abstracts without sufficient data, animal studies, and duplicate reports were excluded.

### 2.4 Inclusion and exclusion criteria

Studies were included if they enrolled participants with a confirmed clinical diagnosis of epilepsy, evaluated an eligible intervention for depressive symptoms or depression-related outcomes, included a comparator group, and reported extractable depression-related outcome data. Only studies published in English were eligible. The primary outcome was depression severity assessed using validated depression rating scales. Baseline depressive symptoms or a formal diagnosis of depression were not required for inclusion if validated depression-related outcomes were reported. Secondary outcomes included clinically significant depressive symptoms, depression incidence, response, and remission. Randomized controlled trials and controlled intervention studies with sufficient data for quantitative synthesis were eligible.

Studies were excluded if they did not include participants with epilepsy, lacked a comparator group, did not report validated depression-related outcomes, provided insufficient data for effect-size calculation, or were case reports, case series, reviews, editorials, letters, protocols, conference abstracts without extractable data, animal studies, or duplicate reports. For multiple publications based on overlapping populations, the most complete or most relevant dataset was included in the primary analysis, while additional follow-up reports were considered in sensitivity analyses when appropriate.

### 2.5 Data sources and search strategy

A comprehensive literature search was conducted in PubMed, Embase, Web of Science, MEDLINE, and Cochrane Library from January 1, 2005 to May 31, 2026. The search strategy combined controlled vocabulary terms and free-text keywords related to epilepsy, depressive symptoms, and eligible interventions. Search terms covered epilepsy and seizure disorders; depression, depressive symptoms, and mood disorders; and intervention-related terms including cognitive behavioral therapy, psychotherapy, psychoeducation, mindfulness, UPLIFT, PEARLS, TIME, SMART, self-management, collaborative care, pharmaceutical care, progressive muscle relaxation, antidepressants, antiseizure medications, transcranial direct current stimulation, and neuromodulation.

The PubMed search strategy included combinations of terms such as “epilepsy,” “seizure,” “depression,” “depressive symptoms,” “major depressive disorder,” “cognitive behavioral therapy,” “psychotherapy,” “mindfulness,” “self-management,” “collaborative care,” “pharmaceutical care,” “progressive muscle relaxation,” “antidepressants,” “antiepileptic drugs,” “antiseizure medications,” and “transcranial direct current stimulation.” The search strategy was adapted for each database according to its specific indexing terms and syntax (Supplementary Table S1).

The reference lists of included studies and relevant systematic reviews were manually screened to identify additional eligible articles. All records were imported into reference management software, and duplicates were removed before screening. Two reviewers independently screened titles and abstracts, followed by full-text assessment of potentially eligible studies. Disagreements were resolved through discussion, and a third reviewer was consulted when consensus could not be reached.

### 2.6 Data extraction and management

Data were independently extracted by two reviewers using a standardized data extraction form in Excel. Extracted information included study characteristics, participant characteristics, intervention and comparator details, depression-related outcome data, effect-size information, and risk-of-bias items.

Study characteristics included first author, publication year, country or region, study design, sample size, number of study arms, follow-up duration, funding information, and trial registration when available. Participant characteristics included age, sex, epilepsy type, seizure frequency, duration of epilepsy, baseline depression status, and baseline depression scale scores when reported. Intervention and comparator information included intervention type, therapeutic category, delivery format, intervention duration, frequency, provider, and comparator type, such as treatment as usual, TAU, care as usual, wait-list control, no intervention, sham control, placebo control, routine antiepileptic treatment, or another active intervention.

Outcome data included continuous depression severity scores measured using validated scales, such as BDI, BDI-II, HAM-D, MADRS, PHQ-9, QIDS, NDDI-E, CES-D, and HSCL-20. Dichotomous depression-related outcomes, including clinically significant depressive symptoms, depression incidence, response, and remission, were also extracted when available. Additional outcomes, such as quality of life, anxiety, adherence, and seizure-related outcomes, were extracted when reported but were not the primary focus of the present analysis.

For effect-size calculation, mean and standard deviation, standard error, confidence interval, change score, event counts, risk ratio, odds ratio, or other convertible estimates were extracted. When standard errors were reported instead of standard deviations, standard deviations were calculated as SD = SE × √n. For continuous depression outcomes, outcome data were extracted according to a prespecified hierarchy. Endpoint depression scores at the analyzed follow-up timepoint were preferred for the primary analysis because they were more consistently reported across the included studies and allowed a uniform comparison across intervention categories. When endpoint scores were unavailable, change-from-baseline scores were extracted. When both endpoint and change scores were available, endpoint scores were used in the primary analysis, and change scores were examined in sensitivity analyses where sufficient data were available.

The analyzed follow-up timepoint was selected according to a prespecified rule. For studies reporting multiple follow-up assessments, the post-intervention assessment closest to 3 months was selected for the primary analysis. When no 3-month assessment was available, the nearest post-treatment timepoint was used. Alternative follow-up assessments were considered in sensitivity analyses when extractable data were available. When multiple validated depression scales were reported in the same study, one prespecified depression scale was selected for the primary analysis to avoid double-counting participants, and alternative scales were considered in sensitivity analyses where appropriate.

Data from multi-arm trials were extracted separately. For pairwise meta-analysis, when more than one eligible intervention arm shared the same control group, the shared comparator group was divided equally across the relevant pairwise comparisons to avoid double-counting participants in pooled analyses. Each intervention arm was then analyzed as an independent comparison. For sensitivity analysis, only the intervention arm most clinically relevant to the primary intervention category was retained when necessary. When multiple publications reported overlapping populations, the most complete or relevant dataset was included in the primary analysis, while additional follow-up reports were considered only in sensitivity analyses. Disagreements during data extraction were resolved by discussion, and unclear or missing information was checked against full-text articles, supplementary materials, and trial registration records when available.

### 2.7 Intervention classification and comparisons

Eligible interventions were classified according to their primary therapeutic component to improve clinical interpretability and reduce heterogeneity within pooled analyses. The main intervention categories were cognitive-behavioral interventions, mindfulness-based interventions, self-management or collaborative-care interventions, relaxation-based interventions, pharmacological interventions, antiseizure medication-related interventions, and neuromodulation-based interventions.

Cognitive-behavioral interventions included Deprexis online CBT, Emyna internet-based CBT, standard CBT, and cognitive-behavioral intervention. Mindfulness-based interventions included Project UPLIFT, mindfulness-based intervention, and mindful breathing training. Self-management or collaborative-care interventions included TIME, SMART, PEARLS, EpINFO, pharmaceutical care, multidisciplinary management, and solution-focused therapy combined with antiepileptic drug treatment. Progressive muscle relaxation was classified as a relaxation-based intervention. Pharmacological interventions included antidepressant comparisons and combined antiseizure medication plus antidepressant regimens. Antiseizure medication-related comparisons included lacosamide, oxcarbazepine, lamotrigine-based regimens, and related active medication comparisons. Neuromodulation-based interventions mainly included active transcranial direct current stimulation compared with sham stimulation.

For pairwise meta-analysis, studies were grouped according to clinically comparable intervention categories and comparator types. The primary comparison was active intervention versus treatment as usual, TAU, wait-list control, no intervention, placebo, sham control, or routine care. Comparisons with at least two studies were quantitatively synthesized. Comparisons supported by only one study were summarized narratively and were not pooled.

### 2.8 Outcomes

The primary outcome was depression severity after intervention, assessed using validated depression rating scales. Eligible scales included BDI, BDI-II, HAM-D, MADRS, PHQ-9, QIDS, NDDI-E, CES-D, HSCL-20, or other validated instruments for depressive symptoms. When multiple validated depression scales were reported within the same study, one prespecified scale was selected for the primary analysis to avoid double counting participants.

Secondary outcomes included dichotomous depression-related outcomes, such as clinically significant depressive symptoms, depression incidence, treatment response, and remission. These outcomes were analyzed separately because adverse depression outcomes and response or remission outcomes have different clinical directions of benefit. For adverse depression outcomes, lower event rates favored the intervention. For response or remission outcomes, higher event rates favored the intervention. Quality of life, anxiety, treatment adherence, and seizure-related outcomes were extracted when available but were not the primary focus of the present meta-analysis.

### 2.9 Statistical analysis

Pairwise meta-analyses were conducted to evaluate the effects of eligible interventions on depression-related outcomes. For continuous depression severity outcomes, standardized mean differences with 95% confidence intervals were calculated because different depression rating scales were used across studies, thereby allowing outcomes measured using different validated instruments and scoring ranges to be synthesized on a common standardized metric. Negative SMD values indicated greater reductions in depression severity in the intervention group compared with the comparator group. For dichotomous outcomes, risk ratios with 95% confidence intervals were calculated from event counts when available. For adverse depression outcomes, RR values less than 1 favored the intervention. For response or remission outcomes, RR values greater than 1 favored the intervention. Random-effects models were used for all primary pooled analyses because clinical and methodological heterogeneity was expected across studies, including differences in intervention content, delivery modality, participant characteristics, baseline depressive symptom severity, follow-up duration, and depression scales. Between-study heterogeneity was quantified using the I^2^ statistic and *τ*^2^. I^2^ values of approximately 25%, 50%, and 75% were interpreted as low, moderate, and high heterogeneity, respectively. Prediction intervals were calculated for pooled analyses including at least three studies to estimate the expected range of intervention effects in a future comparable study. For comparisons including fewer than three studies, prediction intervals were not calculated because of statistical instability.

Standardized mean differences were calculated using either endpoint scores or change-from-baseline scores according to the prespecified hierarchy described above. Endpoint-based SMDs and change-score-based SMDs were not combined within the same subgroup unless their direction and clinical interpretation were consistent. Sensitivity analyses were conducted by replacing endpoint scores with change scores where both were available, or by using alternative depression scales in studies reporting multiple validated depression measures. The robustness of the results was assessed by comparing the direction, magnitude, and statistical significance of the pooled estimates with those of the primary analysis.

The primary analysis focused on active interventions versus control conditions for continuous depression severity. Subgroup analyses were performed according to intervention category when sufficient studies were available, including cognitive-behavioral interventions, mindfulness-based interventions, self-management or collaborative-care interventions, and relaxation-based interventions. Formal tests for subgroup differences were conducted when at least two intervention categories contained two or more studies. Potential effect moderators, including baseline depression severity, delivery modality, intervention dose and duration, outcome scale type, follow-up duration, age group, epilepsy type, epileptogenic zone, seizure frequency, and study quality, were considered descriptively. Formal meta-regression was not performed because the number of studies within each subgroup was small and these moderator variables were inconsistently reported across studies. Additional comparisons, including pharmacological head-to-head comparisons and sham-controlled neuromodulation studies, were summarized separately when they were not sufficiently comparable for pooling with usual-care comparisons. Sensitivity analyses were planned to assess the robustness of the findings. These included excluding studies at high risk of bias, avoiding potential double-counting of overlapping study populations, excluding follow-up reports that may overlap with original trials, using alternative depression scales in studies reporting multiple scales, and comparing random-effects models with fixed-effect models when appropriate. Publication bias and small-study effects were explored using funnel plots when at least ten studies were available for an outcome. Egger's test was not emphasized for subgroup analyses with fewer than ten studies because of limited statistical power.

All analyses were conducted using R or other standard meta-analysis software. Statistical significance was defined using two-sided 95% confidence intervals.

### 2.10 Risk of bias and evidence quality

Risk of bias was assessed independently by two reviewers using the Cochrane Risk of Bias 2 tool for randomized controlled trials. The assessment covered five domains: bias arising from the randomization process, bias due to deviations from intended interventions, bias due to missing outcome data, bias in measurement of the outcome, and bias in selection of the reported result. Each domain was judged as low risk, some concerns, or high risk, and the overall risk of bias for each study was determined according to the RoB 2 guidance. Disagreements were resolved through discussion, and a third reviewer was consulted when necessary.

Because the included quantitative evidence consisted mainly of randomized or controlled intervention studies suitable for RoB 2 assessment, RoB 2 was used as the primary tool for risk-of-bias evaluation. If non-randomized intervention studies were identified but not included in the main quantitative synthesis, they were considered separately and were not pooled with randomized evidence.

The certainty of evidence for the main outcomes was evaluated using the GRADE framework. The assessment considered risk of bias, inconsistency, indirectness, imprecision, and publication bias. Evidence certainty was rated as high, moderate, low, or very low. For comparisons supported by sparse evidence, single-study data, wide confidence intervals, substantial heterogeneity, or concerns regarding study quality, the certainty of evidence was downgraded accordingly. Findings from single studies were interpreted cautiously and were considered insufficient for definitive conclusions. The certainty of evidence for the main continuous depression severity outcomes was assessed using the GRADE framework.

## 3 Result

### 3.1 Study selection

The study selection process is presented in Figure 1. A total of 1,013 records were identified through database searches, including PubMed, MEDLINE, Embase, Cochrane Library, and Web of Science. No additional records were identified from trial registers. Before screening, 16 duplicate records were removed, leaving 997 records for title and abstract screening.

**Figure 1:**
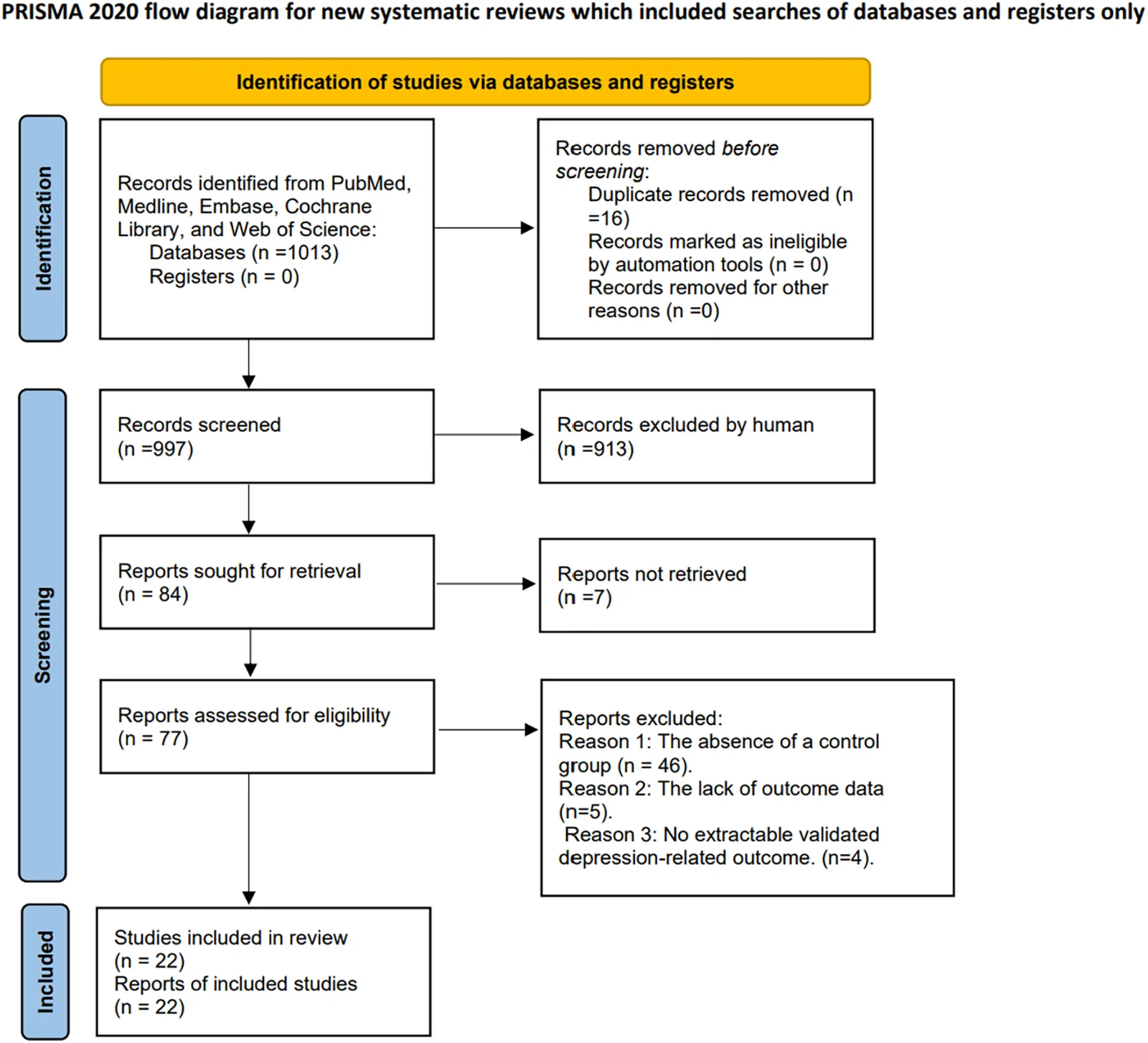
PRISMA 2020 flow diagram.

During title and abstract screening, 913 records were excluded because they were not relevant to the research question, did not involve eligible interventions, did not assess depression-related outcomes, or were not original intervention studies. A total of 84 reports were sought for retrieval, of which 7 reports could not be retrieved. Therefore, 77 full-text reports were assessed for eligibility.

After full-text review, 55 reports were excluded. The main reasons for exclusion were absence of a control group (*n* = 46), lack of extractable outcome data (*n* = 5), and no validated depression-related outcome was reported or extractable (*n* = 4). Finally, 22 studies involving 22 reports met the eligibility criteria and were included in the systematic review and meta-analysis.

Among the included studies, 11 studies contributed data to the primary quantitative synthesis of continuous depression severity. Studies reporting dichotomous depression-related outcomes were analyzed separately according to outcome direction, including adverse depression outcomes and response/remission outcomes. Studies with insufficiently comparable interventions, single-study comparisons, or head-to-head active comparisons that could not be pooled were summarized narratively.

### 3.2 Included studies

#### 3.2.1 Study characteristics

A total of 22 studies were included in the systematic review. The included studies evaluated a wide range of interventions for depressive symptoms in people with epilepsy, including psychological and behavioral interventions, mindfulness-based interventions, self-management or collaborative-care programs, relaxation-based interventions, pharmacological treatments, antiseizure medication-related comparisons, and neuromodulation-based interventions. Most studies were randomized controlled trials, including several sham-controlled or active comparator trials. One study was a three-arm randomized controlled trial. Follow-up durations ranged from short-term assessments at 5 days to long-term follow-up at 18 months.

The included interventions were heterogeneous. Cognitive-behavioral and psychological interventions included Deprexis online CBT, Emyna internet-based CBT, cognitive behavioral intervention, and standard CBT. Mindfulness-based interventions included Project UPLIFT, mindfulness-based intervention, and mindful breathing training. Self-management or collaborative-care interventions included TIME, SMART, PEARLS, EpINFO, pharmaceutical care, multidisciplinary management, and short-term family therapy combined with antiepileptic drugs. Other interventions included progressive muscle relaxation, antidepressant pharmacotherapy, antiseizure medication-related interventions, and transcranial direct current stimulation.

Control conditions included wait-list control, treatment as usual, TAU, care as usual, no-intervention control, AEDs alone, sham stimulation, and active pharmacological comparators. Depression severity was assessed using validated scales, including BDI, BDI-II, HAM-D, MADRS, PHQ-9, QIDS, NDDI-E, CES-D, and HSCL-20. Some studies also reported dichotomous depression-related outcomes, including clinically significant depressive symptoms, depression incidence, response, and remission.

Among the 22 included studies, 11 studies contributed to the primary quantitative synthesis of continuous depression severity. Hum et al. 2019 was a three-arm randomized controlled trial and contributed two eligible comparisons through UPLIFT, EpINFO, and wait-list control. Several studies contributed only to dichotomous depression-related outcomes, whereas pharmacological head-to-head comparisons and sham-controlled neuromodulation studies were summarized separately because they were not clinically comparable with the main pooled comparisons. Detailed characteristics of all included studies are presented in Table 2. Detailed characteristics of the included studies, including intervention modality, intervention dose, depression assessment instruments, baseline depression severity, analyzed follow-up timepoints, and attrition information, are summarized in Supplementary Table S3.

**Table 2:** Study characteristics and role in the evidence synthesis.

Study	Country/Region	Study design	Population	Interventions	Comparator(s)	Sample size	Follow-up	Continuous depression-scale outcome	Role in evidence synthesis
Schröder 2014 (13)	Not specified	Randomized controlled trial	People with epilepsy participating in an online psychological intervention study	Deprexis online cognitive behavioral therapy (CBT)	Wait-list control	38/40	9 weeks	BDI-I total score	Continuous-outcome main analysis
Talo & Turan 2023 (14)	Not specified	Randomized controlled trial	Adults with epilepsy, poor sleep quality (PSQI > 5), and depressive symptoms (BDI > 9)	Progressive muscle relaxation (PMR) exercises	No-intervention control	35/35	4 weeks	BDI	Continuous-outcome main analysis
Sajatovic et al. 2016 (15)	Not specified	Randomized controlled trial	Adults with epilepsy and comorbid serious mental illness	TIME intervention	Treatment as usual (TAU)	19/16 completers at 16 weeks; 22/22 randomized	16 weeks	MADRS; PHQ-9 for sensitivity analysis	Continuous-outcome main analysis/sensitivity analysis
Lai et al. 2021 (16)	Malaysia	Randomized controlled trial	Adults with epilepsy	Mindfulness-based intervention (MBI)	Wait-list control/TAU	14/14; 12-week follow-up 11/14	6 weeks; 12-week follow-up	BDI-II	Continuous-outcome main analysis; follow-up sensitivity analysis
Lim et al. 2024 (17)	Malaysia	Randomized controlled trial	Adults with epilepsy and elevated depressive or anxiety symptoms	20-minute video-guided mindful breathing training	Wait-list control	10/10; 6-week analysis 9/10	2 weeks; 6-week follow-up	NDDI-E	Continuous-outcome main analysis; follow-up sensitivity analysis
Spruill et al. 2021 (18)	USA	Randomized controlled trial	Hispanic adults with epilepsy and elevated depressive symptoms	Project UPLIFT	TAU/epilepsy education	36/36	6 months; 12 months	PHQ-9 continuous score	Continuous-outcome main analysis; dichotomous lower-better analysis/sensitivity analysis
Meyer et al. 2019 (19)	Germany	Randomized controlled trial	Adults with epilepsy	Emyna internet-based CBT plus care as usual (CAU)	Care as usual (CAU)	100/100	3 months	PHQ-9; published SMD/adjusted effect available	Continuous-outcome main analysis; dichotomous response analysis
Sajatovic et al. 2025 (20)	USA	Randomized controlled trial	People with epilepsy receiving self-management/psychosocial intervention	SMART intervention	Wait-list control	80/80	6 months	PHQ-9	Continuous-outcome main analysis
Li et al. 2016 (21)	China	Randomized controlled trial	People with epilepsy	Short-term family therapy (SFT) plus antiepileptic drugs (AEDs)	AEDs alone	52/52	3 months	HAM-D	Continuous-outcome main analysis
Martinović et al. 2006 (22)	Serbia	Randomized controlled trial	Adolescents with epilepsy	Cognitive behavioral intervention (CBI)	TAU/supportive counseling	15/15	9 months	HAM-D; BDI/CES-D for sensitivity analysis	Continuous-outcome main analysis; dichotomous lower-better analysis
Gilliam et al. 2019 (23)	USA	Randomized controlled trial	People with epilepsy and depression	Sertraline	CBT	72/68	16 weeks	BDI; CES-D for sensitivity analysis	Continuous-outcome main analysis; dichotomous response/remission analysis; active head-to-head comparison
Losada-Camacho 2022 (24)	Not specified	Randomized controlled trial/clinical intervention study	People with epilepsy in a pharmaceutical-care study	Pharmaceutical care	TAU	69/74	6 months	Not reported	Dichotomous lower-better main analysis
Zheng 2019 (25)	Not specified	Randomized controlled trial/clinical intervention study	People with epilepsy undergoing multidisciplinary management	Multidisciplinary management	Usual AED treatment/education	92/92	12 months	Not reported	Dichotomous lower-better sensitivity analysis
Thompson 2014 (26)	Not specified	Randomized controlled trial	Adults with epilepsy	Project UPLIFT mindfulness-based CBT	TAU/wait-list control	52/56	10 weeks	Not reported	Dichotomous lower-better main analysis
Ciechanowski 2010 (27)	Not specified	Randomized controlled trial	People with epilepsy and depression	PEARLS depression-care intervention	TAU	35/29	12 months	Not reported	Dichotomous response/remission analysis
Chaytor 2011 (28)	Not specified	Randomized controlled trial/follow-up analysis	People with epilepsy and depression	PEARLS depression-care intervention	TAU	30/28	18 months	Not reported	Dichotomous response sensitivity analysis
Bhatele et al. 2023 (29)	Not specified	Randomized controlled trial	People with epilepsy and mild-to-moderate depression	Escitalopram	Venlafaxine	45/45; per-protocol analysis 42/40	8 weeks	HAM-D	Drug head-to-head comparison; Separate head-to-head comparison
Mota 2021 (30)	Not specified	Randomized sham-controlled trial	People with epilepsy	Home-based tDCS plus TAU	Sham tDCS plus TAU	11/12	60 days	BDI-II	Summarized narratively
Li et al. 2025 (31)	China	Randomized controlled trial	Adolescents aged 12–18 years with focal epilepsy and depression	Lacosamide monotherapy	Oxcarbazepine monotherapy	46/49 completed at 12 months; 53/63 randomized	6 months; 12 months	HAM-D	Antiepileptic-drug head-to-head comparison; Separate head-to-head comparison
Zhang & Zhao 2020 (32)	China	Randomized controlled trial	People with epilepsy and depression	Oxcarbazepine plus escitalopram	Lamotrigine plus escitalopram	53/55	6 months	HAM-D; MADRS for sensitivity analysis	AED plus SSRI head-to-head comparison; Separate head-to-head comparison
Liu et al. 2016 (33)	USA	Randomized sham-controlled trial	Patients with temporal lobe epilepsy	tDCS	Sham stimulation	21/12	5 days; 2 weeks; 4 weeks	BDI change score; NDDI-E change score	Summarized narratively
Hum et al. 2019 (34)	Canada	Three-arm randomized controlled trial	Adults with epilepsy and self-reported depressive symptoms; CESD-R ≥ 12	UPLIFT distance-delivered mindfulness-based CBT; EpINFO distance-delivered epilepsy information/self-management	Wait-list control (WLC)	UPLIFT 20/EpINFO 24/WLC 11	8 weeks; 6 months; 1 year	QIDS for primary analysis; NDDI-E for sensitivity/supplementary analysis	Three-arm trial; UPLIFT and EpINFO were separately compared with WLC, with shared-control adjustment.

#### 3.2.2 Intervention classification and contribution to evidence synthesis

The included interventions were classified according to their primary therapeutic components to improve clinical interpretability and to reduce heterogeneity within pooled analyses. The main intervention categories included cognitive-behavioral interventions, mindfulness-based interventions, self-management or collaborative-care interventions, relaxation-based interventions, pharmacological interventions, antiseizure medication-related interventions, and neuromodulation-based interventions.

Cognitive-behavioral interventions included Deprexis online CBT, Emyna internet-based CBT, standard CBT, and cognitive-behavioral intervention. Mindfulness-based interventions included Project UPLIFT, mindfulness-based intervention, and mindful breathing training. Self-management or collaborative-care interventions included TIME, SMART, PEARLS, EpINFO, pharmaceutical care, multidisciplinary management, and solution-focused therapy combined with antiepileptic drug treatment. Progressive muscle relaxation was classified as a relaxation-based intervention. Pharmacological and antiseizure medication-related interventions included antidepressant comparisons, antiseizure medication monotherapy comparisons, and combined antiseizure medication plus antidepressant regimens. Neuromodulation-based interventions mainly included active transcranial direct current stimulation compared with sham stimulation.

For the primary continuous outcome of depression severity, 11 studies contributed data to the quantitative synthesis. These studies were grouped according to intervention category and comparator type. CBT was supported by three studies, mindfulness-based interventions by four studies, self-management or collaborative-care interventions by four studies, and progressive muscle relaxation by one study. Because progressive muscle relaxation was supported by only one trial, this comparison was summarized descriptively and was interpreted cautiously. Hum et al. 2019 was a three-arm randomized controlled trial and contributed two eligible intervention comparisons, UPLIFT versus wait-list control and EpINFO versus wait-list control; the shared control group was handled appropriately to avoid double-counting participants in pooled analyses. (Table 3)

**Table 3:** Domain-level risk of bias assessment of the included studies using the cochrane RoB 2 tool.

Study	Randomization process	Deviations from intended interventions	Missing outcome data	Measurement of the outcome	Selection of the reported result	Overall risk of bias	Primary reason/comments
Schröder 2014 (13)	Some concerns	Some concerns	Some concerns	Some concerns	Some concerns	Some concerns	Online psychological intervention versus wait-list control; participant blinding was not feasible and depression was self-reported. Check allocation concealment and prespecified analysis.
Talo & Turan 2023 (14)	Some concerns	Some concerns	Low	Some concerns	Some concerns	Some concerns	PMR versus no intervention; performance/placebo effects and self-reported BDI may influence estimates. Randomization details should be checked in the full text.
Sajatovic et al. 2016 (15)	Some concerns	Some concerns	Some concerns	Some concerns	Some concerns	Some concerns	Small RCT with completer numbers lower than randomized numbers; self-reported/clinical depression measures used in an unblinded psychosocial intervention.
Lai et al. 2021 (16)	Some concerns	Some concerns	Some concerns	Some concerns	Some concerns	Some concerns	Mindfulness intervention versus wait-list/TAU; small sample and incomplete follow-up. Participant blinding was not feasible.
Lim et al. 2024 (17)	Some concerns	Some concerns	Some concerns	Some concerns	Some concerns	Some concerns	Very small wait-list controlled trial; depression was assessed using self-report scale. Follow-up attrition should be verified.
Spruill et al. 2021 (18)	Some concerns	Some concerns	Some concerns	Some concerns	Some concerns	Some concerns	UPLIFT-type intervention with usual-care/education comparator; self-reported PHQ-9 and possible attrition at longer follow-up.
Meyer et al. 2019 (19)	Low	Some concerns	Some concerns	Some concerns	Some concerns	Some concerns	Large RCT of internet-based CBT; self-report PHQ-9 and incomplete follow-up can raise some concerns despite stronger design.
Sajatovic et al. 2025 (20)	Some concerns	Some concerns	Some concerns	Some concerns	Some concerns	Some concerns	Self-management/psychosocial intervention with wait-list control; outcome measurement and attrition require confirmation from full text.
Li et al. 2016 (21)	Some concerns	Some concerns	Some concerns	Some concerns	Some concerns	Some concerns	Family therapy plus AEDs versus AEDs alone; blinding likely not feasible and randomization/allocation procedures need confirmation.
Martinović et al. 2006 (22)	Some concerns	Some concerns	Some concerns	Some concerns	Some concerns	Some concerns	Small adolescent trial with multiple depression scales; older trial reporting may not fully describe allocation concealment or prespecified outcomes.
Gilliam et al. 2019 (23)	Low	Some concerns	Some concerns	Some concerns	Low	Some concerns	Active head-to-head trial of sertraline versus CBT; participant blinding not feasible and depression outcomes include self-report measures.
Losada-Camacho 2022 (24)	Some concerns	Some concerns	Some concerns	Some concerns	Some concerns	Some concerns	Pharmaceutical-care intervention; confirm randomization/allocation procedures and whether CES-D endpoint reporting was prespecified.
Zheng 2019 (25)	Some concerns	Some concerns	Some concerns	Some concerns	Some concerns	Some concerns	Multidisciplinary management study; reported dichotomous depression endpoint requires checking for endpoint definition and selective reporting.
Thompson 2014 (26)	Some concerns	Some concerns	Low	Some concerns	Some concerns	Some concerns	UPLIFT versus TAU/wait-list; depression diagnosis/event outcome may be less prone than self-report scales, but blinding and reporting details require confirmation.
Ciechanowski 2010 (27)	Low	Some concerns	Some concerns	Some concerns	Low	Some concerns	PEARLS depression-care intervention; generally randomized design, but psychosocial intervention was unblinded and attrition should be checked.
Chaytor 2011 (28)	Some concerns	Some concerns	High	Some concerns	Some concerns	High	Longer-term follow-up/secondary analysis of PEARLS; possible attrition and overlap with the original trial make this less suitable for the primary quantitative synthesis.
Bhatele et al. 2023 (29)	Low	Some concerns	Some concerns	Low	Low	Some concerns	Drug head-to-head RCT; outcome scales and response/remission endpoints were clear, but blinding/open-label status and per-protocol losses should be verified.
Mota 2021 (30)	Some concerns	Low	Some concerns	Low	Some concerns	Some concerns	Sham-controlled tDCS trial; blinding is more feasible, but sample size is small and attrition/reporting details should be checked.
Li et al. 2025 (31)	Some concerns	Some concerns	Some concerns	Some concerns	Some concerns	Some concerns	Antiepileptic-drug head-to-head trial in adolescents; randomization/blinding and missing data need verification.
Zhang & Zhao 2020 (32)	Some concerns	Some concerns	Some concerns	Some concerns	Some concerns	Some concerns	Head-to-head drug-combination trial; allocation concealment, blinding, and prespecified outcome reporting should be checked.
Liu et al. 2016 (33)	Some concerns	Low	Some concerns	Low	Some concerns	Some concerns	Sham-controlled tDCS trial with short follow-up; blinding feasible, but small sample and change-score reporting require caution.
Hum et al. 2019 (34)	Low	Some concerns	Some concerns	Some concerns	Low	Some concerns	Three-arm RCT with stratified randomization. Results were reported as mean ± SE and per-protocol analyses; self-rated QIDS/NDDI-E outcomes were used.

Several studies did not contribute to the primary continuous-outcome meta-analysis but provided additional evidence for dichotomous depression-related outcomes, pharmacological head-to-head comparisons, or sham-controlled neuromodulation comparisons. These comparisons were summarized separately when they were not clinically comparable with usual-care, wait-list, or no-intervention control comparisons, or when only one study was available for a given comparison. This approach was used to ensure that quantitative pooling was restricted to clinically and methodologically comparable studies.

#### 3.2.3 Risk of bias assessment

Risk of bias was assessed using the Cochrane RoB 2 tool. Among the 22 included studies, none was judged to be at low risk of bias overall, 21 studies were judged as having some concerns, and 1 study was judged to be at high risk of bias. The only study rated as high risk was Chaytor 2011, mainly because of substantial missing outcome data during long-term follow-up and potential overlap with the original PEARLS trial population.

Across the five RoB 2 domains, the most common concerns were related to deviations from intended interventions, measurement of the outcome, and missing outcome data. Because most psychological, behavioral, mindfulness-based, and self-management interventions were open-label by nature, blinding of participants and personnel was generally not feasible. In addition, depression outcomes were frequently assessed using self-reported scales, such as BDI, PHQ-9, QIDS, NDDI-E, and related instruments, which raised some concerns in the measurement-of-outcome domain. Several studies also had incomplete follow-up or completer-based analyses, leading to some concerns regarding missing outcome data.

In the randomization process domain, several studies were rated as low risk, including Meyer et al. 2019, Gilliam et al. 2019, Ciechanowski 2010, Bhatele et al. 2023, and Hum et al. 2019. Most other studies were rated as having some concerns because sequence generation or allocation concealment was insufficiently described. In the domain of selection of the reported result, a few studies were judged as low risk, whereas most were rated as having some concerns because trial protocols or prespecified analysis plans were not clearly available.

Overall, the risk-of-bias assessment suggests that the evidence base should be interpreted cautiously. The main limitations were the open-label nature of many interventions, reliance on self-reported depression outcomes, incomplete outcome data in some trials, and limited reporting of prespecified outcomes. Detailed domain-level judgments for each study are presented in Table 2, and the overall distribution of RoB 2 judgments is shown in Figure 2.

**Figure 2:**
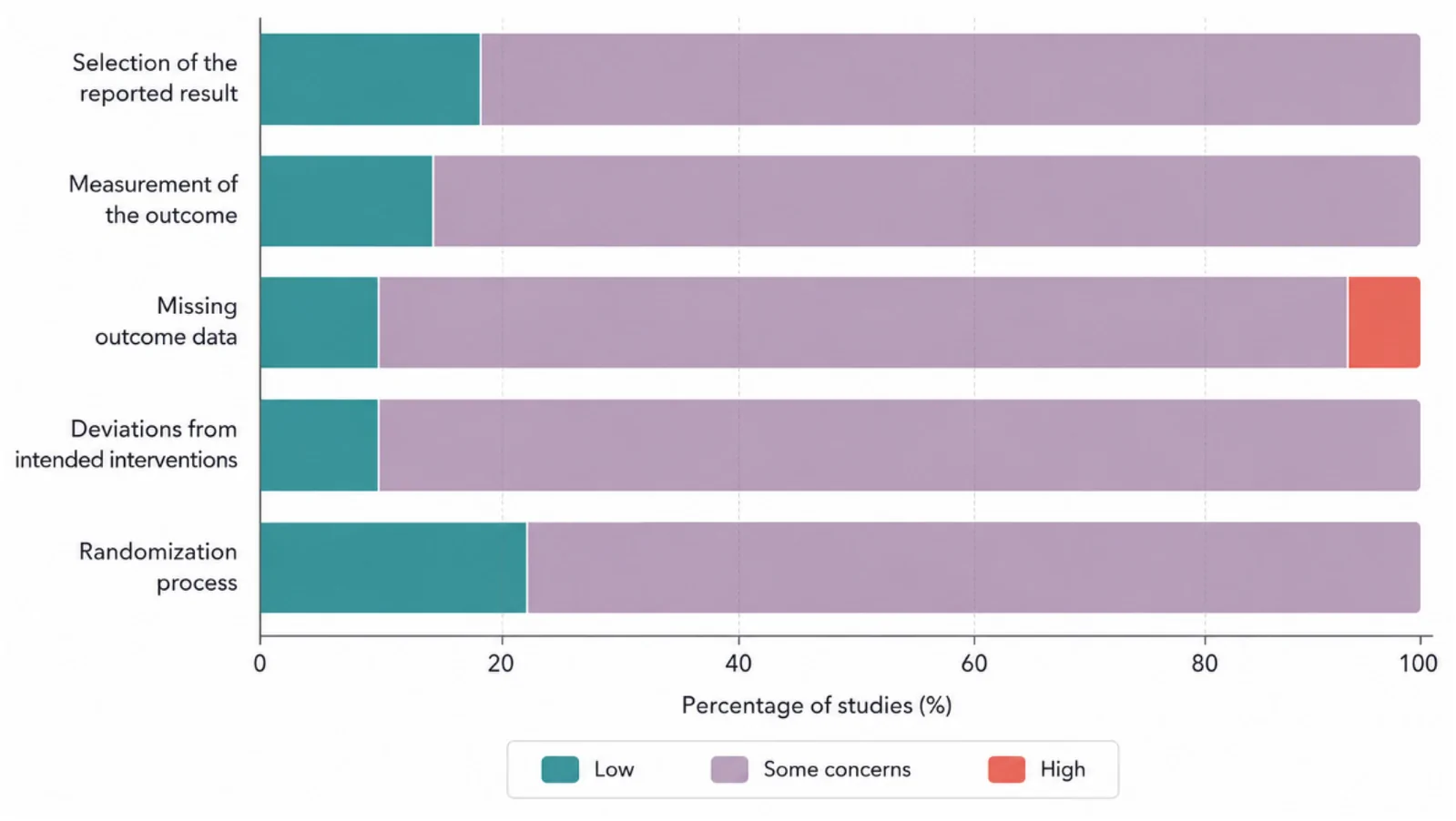
Risk-of-bias summary plot according to the cochrane RoB 2 tool.

#### 3.2.4 Intervention classification

Interventions were classified according to their primary therapeutic component to improve clinical interpretability and reduce heterogeneity within pooled analyses. Cognitive-behavioral interventions included Deprexis online CBT, Emyna internet-based CBT, standard cognitive behavioral therapy, and cognitive-behavioral intervention. Mindfulness-based interventions included Project UPLIFT, mindfulness-based intervention, and mindful breathing training. Self-management, collaborative-care, and care-based interventions included TIME, SMART, PEARLS, EpINFO, multidisciplinary management, pharmaceutical care, and solution-focused therapy delivered alongside antiepileptic drug treatment. Progressive muscle relaxation was classified as a relaxation-based intervention.

Pharmacological and neuromodulation-based interventions were classified separately. Antidepressant pharmacotherapy included sertraline, escitalopram, and venlafaxine. Antiseizure medication-related comparisons included lacosamide, oxcarbazepine, and combined antiseizure medication plus antidepressant regimens. Neuromodulation-based interventions mainly included active transcranial direct current stimulation compared with sham stimulation.

Control conditions included treatment as usual, TAU, care as usual, wait-list control, no-intervention control, routine antiepileptic drug treatment or education, placebo control, and sham control. For the primary quantitative synthesis, clinically similar control conditions were grouped as TAU/control when appropriate. Comparisons were pooled only when intervention categories and comparator conditions were clinically comparable. Comparisons supported by a single study, active head-to-head pharmacological comparisons, and sham-controlled neuromodulation comparisons were summarized separately when quantitative pooling was not appropriate. Chaytor 2011 was considered only in sensitivity analysis because it may overlap with the PEARLS trial population reported by Ciechanowski 2010 (Table 4).

**Table 4:** Classification of interventions and comparators included in the evidence synthesis.

Intervention category	Specific interventions	No. of studies	Role in evidence synthesis
CBT/cognitive-behavioral interventions	Deprexis online CBT; Emyna internet-based CBT; standard CBT; cognitive-behavioral intervention	4	Included in continuous depression severity synthesis; dichotomous response/remission data summarized when available
MBCT/mindfulness-based interventions	UPLIFT; mindfulness-based intervention; mindful breathing training	5	Included in continuous depression severity synthesis; UPLIFT-related dichotomous outcomes summarized separately
Self-management/collaborative care/education	TIME; SMART; PEARLS; EpINFO; multidisciplinary management; pharmaceutical care; solution-focused therapy plus AEDs	7 main; 8 including Chaytor 2011 sensitivity	Included in continuous and/or dichotomous outcome synthesis according to available outcome data; Chaytor 2011 considered only in sensitivity analysis
Relaxation exercise	Progressive muscle relaxation	1	Single-study evidence for continuous depression severity; summarized descriptively
Antidepressant pharmacotherapy	Sertraline; escitalopram; venlafaxine; oxcarbazepine plus escitalopram; lamotrigine plus escitalopram	3	Active head-to-head pharmacological comparisons; summarized separately
AED-based pharmacological interventions	AEDs alone; lacosamide; oxcarbazepine; oxcarbazepine plus escitalopram; lamotrigine plus escitalopram	3	Antiseizure medication-related comparisons; summarized separately
tDCS/neuromodulation	Active tDCS; sham tDCS comparator	2	Sham-controlled neuromodulation comparisons; summarized separately
Control/TAU/TAU/wait-list	TAU; TAU; CAU; wait-list; no intervention; routine AED care; placebo control; sham control	16 control arms; 15 excluding Chaytor 2011	Comparator category for quantitative or descriptive synthesis, depending on clinical comparability

### 3.3 Studies included in the continuous-outcome meta-analysis

Table 5 summarizes the studies and sample sizes included in the quantitative synthesis of continuous depression severity outcomes. The primary analysis focused on comparisons between active psychological and behavioral interventions and treatment as usual (TAU)/control conditions.

**Table 5:** Studies contributing to the continuous depression severity meta-analysis.

Direct comparison	No. of studies	Sample size	Direct effect, SMD (95% CI)
PMR vs. TAU/control	k = 1	*N* = 70	−1.65 (-2.32, −0.98)
CBT vs. TAU/control	k = 3	*N* = 308	−0.55 (−0.90, −0.21)
MBCT/mindfulness vs. TAU/control	k = 4	*N* = 151	−0.46 (−0.86, −0.07)
Self-management/care vs. TAU/control	k = 4	*N* = 334	−0.43 (−0.74, −0.12)
UPLIFT vs. TAU/control	k = 2	*N* = 103	Included in MBCT/mindfulness
Emyna vs. TAU/control	k = 1	*N* = 200	Included in CBT
TIME vs. TAU/control	k = 1	*N* = 35	Included in self-management/care
SMART vs. TAU/control	k = 1	*N* = 160	Included in self-management/care
EpINFO vs. TAU/control	k = 1	*N* = 35	Included in self-management/care
CBI vs. TAU/control	k = 1	*N* = 30	Included in CBT
SFT + AEDs vs. AED alone/routine care	k = 1	*N* = 104	Included in self-management/care

Most specific intervention comparisons were supported by single-study evidence. Therefore, although the pooled category-level estimates suggested favorable effects of active interventions compared with TAU/control, the results should be interpreted cautiously, particularly for PMR, which was supported by only one small trial.

At the intervention-category level, cognitive behavioral therapy (CBT) was supported by three studies, mindfulness-based interventions by four studies, self-management or collaborative-care interventions by four studies, and progressive muscle relaxation (PMR) by one study. At the intervention-specific level, only the UPLIFT versus TAU/control comparison was supported by more than one study, whereas most other specific intervention comparisons were derived from single studies.

Negative standardized mean difference (SMD) values indicate greater reductions in depressive symptom severity in the intervention group compared with TAU/control. For intervention categories supported by at least two studies, random-effects pairwise meta-analyses were performed. In contrast, comparisons supported by a single study, such as PMR versus TAU/control, were summarized descriptively and interpreted with caution because of limited evidence.

For multi-arm trials, shared control groups were appropriately adjusted to avoid double-counting participants in pooled analyses. Overall, although category-level pooled estimates suggested beneficial effects of active psychological and behavioral interventions on depressive symptoms compared with TAU/control, the certainty of evidence varied across intervention types. In particular, findings based on single-study evidence should be interpreted cautiously.

### 3.4 Continuous depression severity outcome

#### 3.4.1 Study-level effects by intervention category

For the primary continuous outcome of depression severity, 12 active-versus-control comparisons from 11 studies were included in the quantitative synthesis. Because different validated depression rating scales were used across studies, the standardized mean difference was selected as the effect measure. Negative SMD values indicate greater reductions in depression severity in the active intervention group compared with TAU/control.

The study-level results are summarized by intervention category in Table 6. Three comparisons were classified as CBT or cognitive-behavioral interventions, four as mindfulness-based interventions, four as self-management or collaborative-care interventions, and one as a relaxation-based intervention. Most study-level estimates favored active psychological and behavioral interventions over TAU/control, although the magnitude and statistical significance of effects varied across studies.

**Table 6:** Study-level effect estimates for continuous depression severity by intervention category.

Intervention category	Study	Specific comparison	*n* intervention	*n* control	SMD	95% CI	Interpretation
CBT/cognitive-behavioral interventions	Schröder 2014	Deprexis online CBT vs. wait-list control	38	40	−0.217	−0.662 to 0.228	Direction favored CBT, but not statistically significant
CBT/cognitive-behavioral interventions	Meyer et al. 2019	Emyna eCBT + CAU vs. CAU	100	100	−0.540	−0.825 to −0.255	Significant improvement favoring CBT
CBT/cognitive-behavioral interventions	Martinović et al. 2006	CBI vs. TAU	15	15	−1.364	−2.164 to −0.564	Significant improvement favoring CBT
MBCT/mindfulness-based interventions	Lai et al. 2021	MBI vs. wait-list control	14	14	−0.656	−1.418 to 0.106	Direction favored mindfulness, but not statistically significant
MBCT/mindfulness-based interventions	Lim et al. 2024	Mindful breathing vs. wait-list control	10	10	−1.069	−2.013 to −0.126	Significant improvement favoring mindfulness
MBCT/mindfulness-based interventions	Spruill et al. 2021	UPLIFT vs. TAU	36	36	−0.277	−0.741 to 0.188	Direction favored mindfulness, but not statistically significant
MBCT/mindfulness-based interventions	Hum et al. 2019	UPLIFT vs. wait-list control	20	5.5*	−0.232	−1.178 to 0.714	Direction favored UPLIFT, but not statistically significant
Self-management/collaborative-care interventions	Sajatovic 2016	TIME vs. TAU	19	16	−1.071	−1.783 to −0.360	Significant improvement favoring self-management/care
Self-management/collaborative-care interventions	Sajatovic et al. 2025	SMART vs. wait-list control	80	80	−0.250	−0.561 to 0.061	Direction favored self-management/care, but not statistically significant
Self-management/collaborative-care interventions	Li et al. 2016	SFT + AEDs vs. AEDs alone	52	52	−0.558	−0.950 to −0.166	Significant improvement favoring self-management/care
Self-management/collaborative-care interventions	Hum et al. 2019	EpINFO vs. wait-list control	24	5.5*	−0.020	−0.946 to 0.907	No clear difference
Relaxation-based intervention	Talo & Turan 2023	PMR vs. no intervention	35	35	−1.667	−2.211 to −1.122	Large significant improvement, but single-study evidence

Among CBT-related interventions, Meyer et al. 2019 and Martinović et al. 2006 showed statistically significant reductions in depression severity, whereas Schröder 2014 showed a favorable but non-significant direction of effect. Among mindfulness-based interventions, Lim et al. 2024 showed a statistically significant benefit, while Lai et al. 2021, Spruill et al. 2021, and Hum et al. 2019 showed favorable but imprecise estimates. Among self-management or collaborative-care interventions, Sajatovic et al. 2016 and Li et al. 2016 showed statistically significant benefits, whereas SMART and EpINFO showed non-significant effects. Progressive muscle relaxation showed the largest study-level effect, but this finding was based on a single small trial and should therefore be interpreted cautiously.

Hum et al. 2019 was a three-arm randomized controlled trial and contributed two eligible comparisons, UPLIFT versus wait-list control and EpINFO versus wait-list control. In the overall analysis, the shared wait-list control group was split to avoid double-counting participants.

#### 3.4.2 Pairwise meta-analysis of active interventions versus TAU/control

The forest plot of active interventions versus TAU/control is shown in Figure 3. Random-effects pairwise meta-analysis was used because clinical and methodological heterogeneity was expected across studies, including differences in intervention content, delivery format, participant characteristics, depression scales, and follow-up duration.

**Figure 3:**
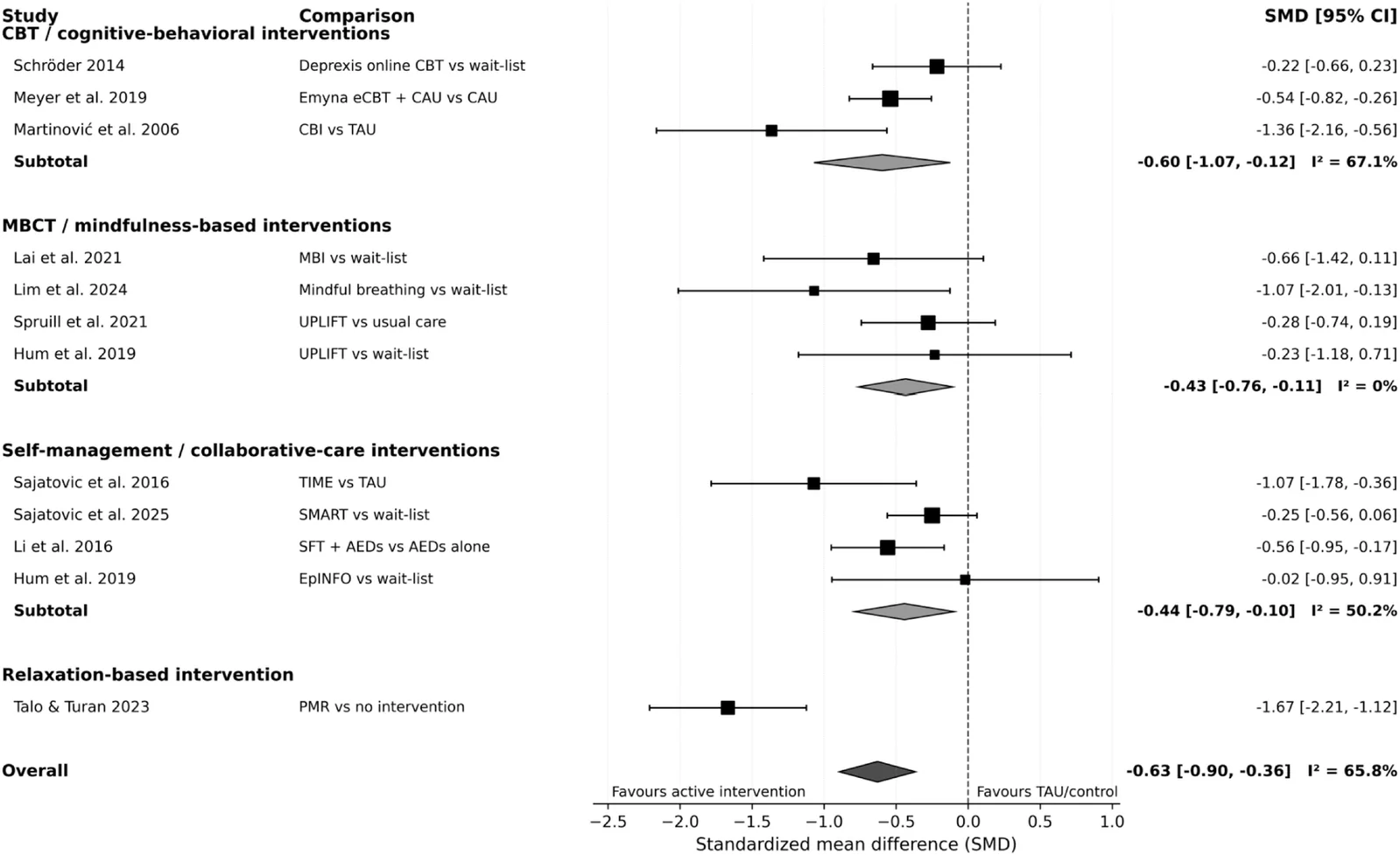
Forest plot of active interventions versus TAU/control for continuous depression severity. Standardized mean differences with 95% confidence intervals are shown. Negative SMD values favor the active intervention over TAU/control. TAU/control included TAU, treatment as usual, care as usual, wait-list control, no-intervention control, or routine antiepileptic treatment when clinically appropriate. For the three-arm study by Hum et al. 2019, the shared wait-list control group was split in the overall analysis to avoid double-counting participants.

Overall, active interventions were associated with greater reductions in depression severity compared with TAU/control (SMD = −0.63, 95% CI: −0.90 to −0.36). Moderate to substantial heterogeneity was observed and is reported in detail in Section 6. In subgroup analyses, CBT, MBCT/mindfulness-based interventions, and self-management/care interventions all showed favorable pooled effects compared with TAU/control. Progressive muscle relaxation showed a large effect in a single trial but was not pooled as an independent subgroup because only one study was available. Therefore, the PMR result should be considered exploratory rather than definitive. Given the heterogeneity of intervention types, subgroup estimates by intervention category were considered more clinically informative than the overall pooled estimate.

#### 3.4.3 Comparative magnitude of effects across intervention categories

Across the analyzed intervention categories, all active interventions showed greater reductions in depressive symptom severity than TAU/control, although the magnitude and certainty of the effects varied. PMR showed the largest estimated effect (SMD = −1.65, 95% CI: −2.32 to −0.98), but this finding was based on only one small trial and should therefore be interpreted cautiously. CBT showed a moderate and statistically significant effect (SMD = −0.55, 95% CI: −0.90 to −0.21). MBCT/mindfulness-based interventions (SMD = −0.46, 95% CI: −0.86 to −0.07) and self-management/care interventions (SMD = −0.43, 95% CI: −0.74 to −0.12) also showed significant small-to-moderate effects. Compared with PMR, the estimates for CBT, MBCT/mindfulness-based interventions, and self-management/care interventions were supported by multiple studies and may therefore provide more clinically reliable evidence (Table 7 and Figure 4).

**Table 7:** Comparative magnitude of effects by intervention category.

Intervention category	No. of studies	Sample size	SMD	95% CI	Evidence interpretation
PMR	1	70	−1.65	−2.32 to −0.98	Largest effect estimate; single-study evidence
CBT	3	308	−0.55	−0.90 to −0.21	Moderate effect; supported by multiple studies
MBCT/mindfulness	4	151	−0.46	−0.86 to −0.07	Small-to-moderate effect
Self-management/care	4	334	−0.43	−0.74 to −0.12	Small-to-moderate effect; supported by multiple studies

**Figure 4:**
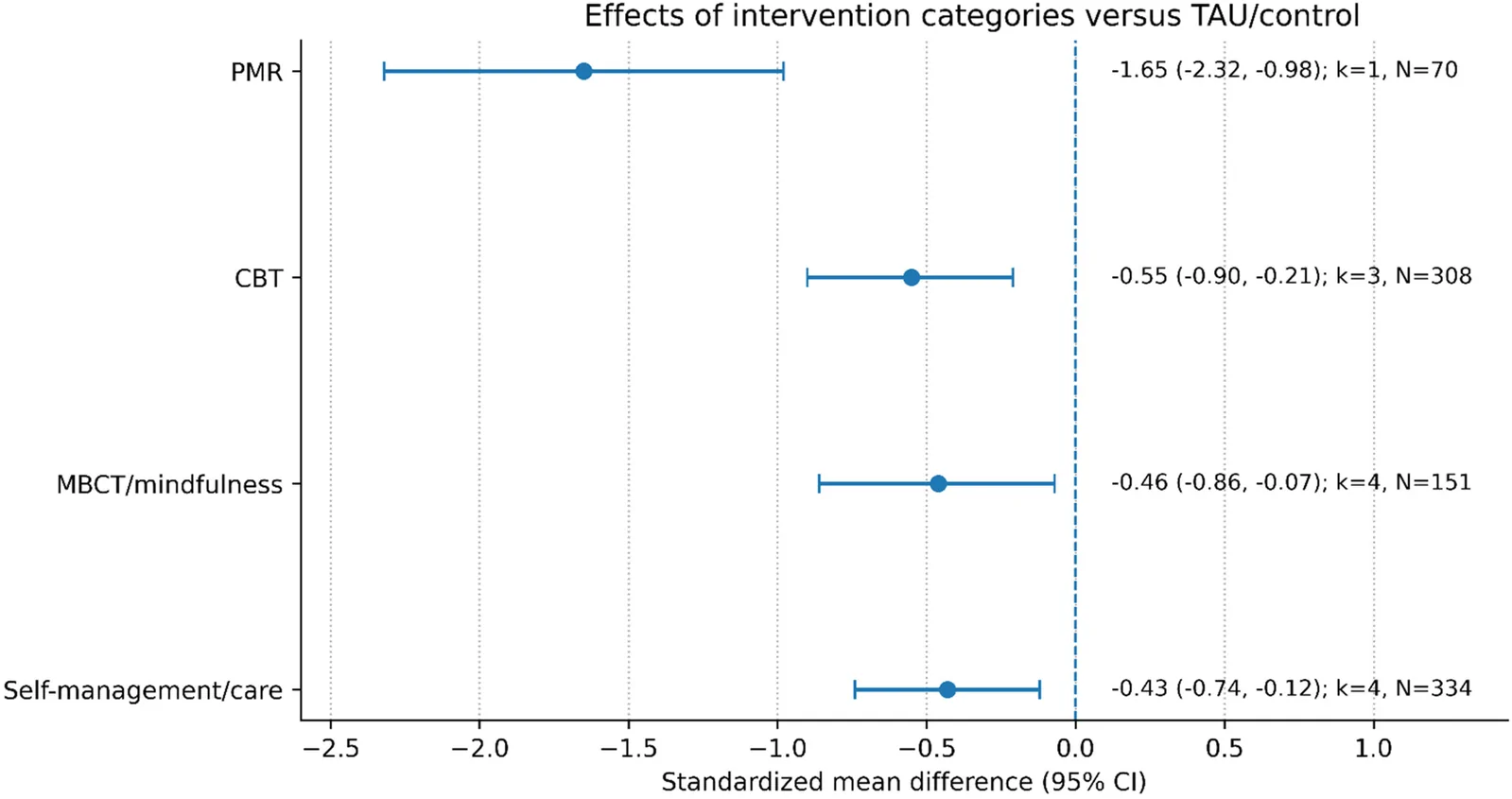
Forest plot of pooled effects by intervention category versus TAU/control for depression severity. The figure shows standardized mean differences (SMDs) and 95% confidence intervals (CIs) for depressive symptom severity across intervention categories compared with TAU/control. Negative SMD values indicate greater reductions in depressive symptoms in the intervention group. PMR showed the largest estimated effect, but this estimate was based on a single small trial and should be interpreted cautiously.

### 3.5 Secondary outcomes

#### 3.5.1 Dichotomous depression-related outcomes

Dichotomous depression-related outcomes were analyzed separately from the primary continuous outcome because their definitions and directions of benefit differed. These outcomes were classified into adverse depression outcomes and response/remission outcomes. For adverse depression outcomes, including depression incidence, clinically significant depressive symptoms, and moderate-to-severe depression, RR values less than 1 favored the intervention. For response/remission outcomes, including depression response, remission, and clinically significant improvement, RR values greater than 1 favored the intervention.

For adverse dichotomous depression outcomes, pharmaceutical care significantly reduced the proportion of patients with clinically significant depressive symptoms compared with TAU (RR = 0.64, 95% CI: 0.42 to 0.99) (24). UPLIFT (26) and cognitive-behavioral intervention (22) showed favorable point estimates, but their confidence intervals were wide and crossed the null value. Multidisciplinary management also showed a favorable but non-significant effect compared with routine AED care (25).

For response/remission outcomes, Emyna plus care as usual significantly increased the probability of clinically significant improvement compared with care as usual alone (RR = 1.93, 95% CI: 1.11 to 3.38) (19). PEARLS showed favorable but imprecise effects on depression response (27). Chaytor 2011 was considered sensitivity evidence because it was a long-term follow-up analysis of the PEARLS trial and may have overlapped with the population reported by Ciechanowski 2010 (28). Active head-to-head comparisons, including escitalopram versus venlafaxine (29) and sertraline versus CBT (23), did not show clear differences in response or remission. (Table 8 and Figure 5)

**Table 8:** Effects of interventions on adverse dichotomous depression outcomes.

Outcome category	Study	Comparison	Outcome	RR (95% CI)
Adverse depression outcome	Losada-Camacho 2022 (24)	Pharmaceutical care vs. TAU	CES-D depression rate ≥20	0.64 (0.42 to 0.99)
Adverse depression outcome	Thompson 2014 (26)	UPLIFT vs. TAU/wait-list	MDD incidence/new or relapse	0.08 (0.00 to 1.43)
Adverse depression outcome	Martinović 2006 (22)	CBI vs. TAU	Depression incidence	0.14 (0.01 to 2.55)
Adverse depression outcome	Zheng 2019 (25)	Multidisciplinary management vs. routine AED care	Moderate-to-severe depression	0.83 (0.38 to 1.83)
Response/remission outcome	Ciechanowski 2010 (27)	PEARLS vs. TAU	≥50% depression response	3.31 (0.76 to 14.40)
Response/remission outcome	Chaytor 2011 (28)	PEARLS vs. TAU	>40% symptom reduction	3.11 (0.95 to 10.15)
Response/remission outcome	Meyer 2019 (19)	Emyna + CAU vs. CAU	Clinically significant improvement	1.93 (1.11 to 3.38)
Response/remission outcome	Bhatele 2023 (29)	Escitalopram vs. venlafaxine	Response ≥50% HAM-D improvement	1.01 (0.84 to 1.21)
Response/remission outcome	Bhatele 2023 (29)	Escitalopram vs. venlafaxine	Remission HAM-D < 7	1.02 (0.78 to 1.32)
Response/remission outcome	Gilliam 2019 (23)	Sertraline vs. CBT	MINI depression remission	0.88 (0.65 to 1.17)

For adverse depression outcomes, RR < 1 favors the intervention. For response/remission outcomes, RR > 1 favors the intervention. Chaytor 2011 was treated as sensitivity evidence because of potential overlap with the PEARLS population reported by Ciechanowski 2010. Active head-to-head comparisons were summarized descriptively. Due to heterogeneity in outcome definitions and comparator types, dichotomous outcomes were not pooled into a single overall estimate.

**Figure 5:**
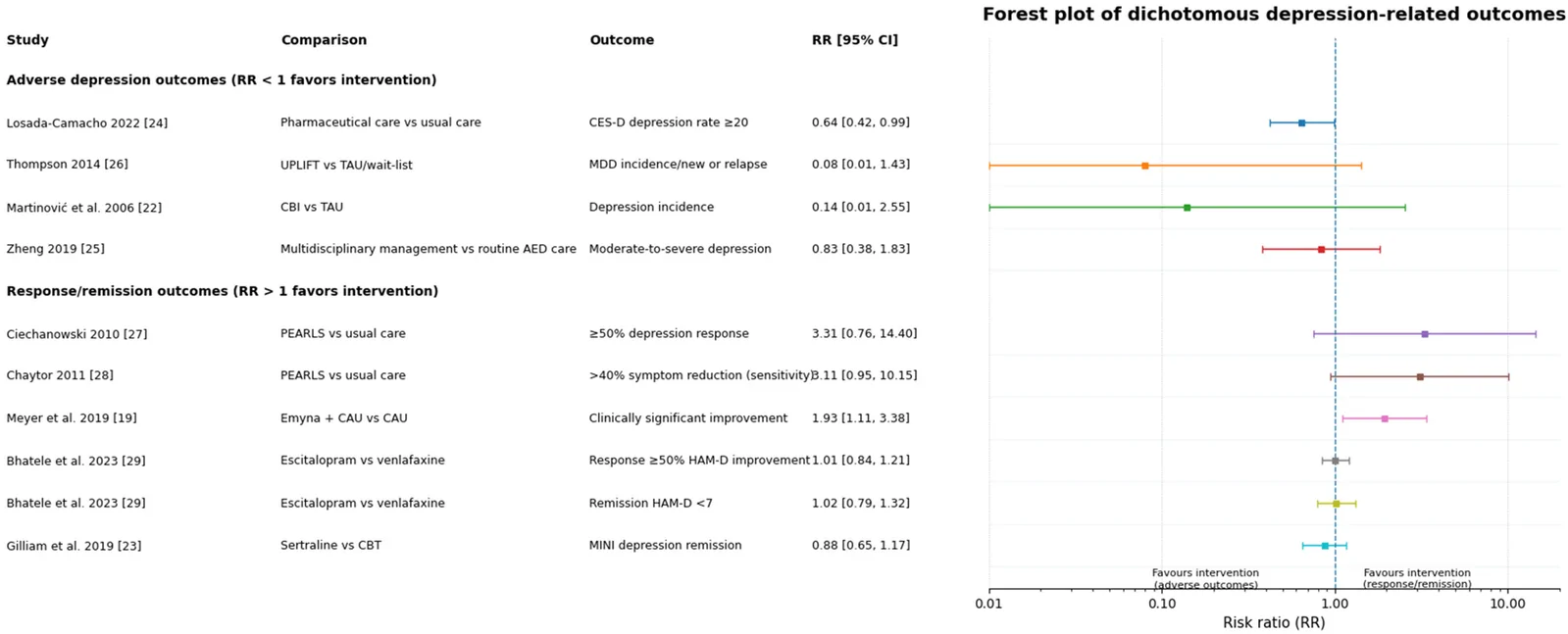
Forest plot of adverse dichotomous depression outcomes.

Overall, the dichotomous outcome evidence was sparse and most comparisons were informed by single studies. Because outcome definitions, comparator types, and directions of benefit differed across studies, these outcomes were summarized descriptively rather than pooled into a single overall estimate. Therefore, the dichotomous findings should be interpreted as supportive secondary evidence rather than definitive pooled evidence.

### 3.6 Heterogeneity, sensitivity, and subgroup analyses

Heterogeneity was assessed separately within each pooled pairwise comparison using I^2^ and *τ*^2^ statistics. For the overall analysis of active psychological and behavioral interventions versus TAU/control, heterogeneity was moderate to substantial (I^2^ = 65.8%, *τ*^2^ = 0.127). The 95% prediction interval ranged from −1.48 to 0.22, suggesting that the magnitude of effect may vary substantially across future comparable studies.

In subgroup analyses, CBT or cognitive-behavioral interventions showed moderate to substantial heterogeneity (I^2^ = 67.1%, *τ*^2^ = 0.113), with a wide 95% prediction interval of −5.86 to 4.66. This prediction interval should be interpreted cautiously because only three studies contributed to this subgroup. Mindfulness-based interventions showed no observed statistical heterogeneity (I^2^ = 0%, *τ*^2^ = 0.000), with a 95% prediction interval of −1.15 to 0.29. Self-management or collaborative-care interventions showed moderate heterogeneity (I^2^ = 50.2%, *τ*^2^ = 0.059), with a 95% prediction interval of −1.73 to 0.85. PMR was supported by only one study, and heterogeneity could not be estimated (Table 9).

**Table 9:** Heterogeneity statistics and prediction intervals for continuous depression severity.

Analysis	No. of contrasts	I^2^	*τ* ^2^	95% prediction interval
Overall active interventions vs. TAU/control	12	65.8%	0.127	−1.48 to 0.22
CBT/cognitive-behavioral interventions	3	67.1%	0.113	−5.86 to 4.66
MBCT/mindfulness-based interventions	4	0%	0.000	−1.15 to 0.29
Self-management/care interventions	4	50.2%	0.059	−1.73 to 0.85
PMR	1	Not estimable	Not estimable	Not estimable

Sensitivity analyses were conducted to assess the robustness of the main findings. Chaytor 2011, a long-term follow-up analysis of the PEARLS trial, was not included in the main analysis to avoid potential participant overlap with Ciechanowski 2010 and was considered only as sensitivity evidence. Active head-to-head pharmacological comparisons and sham-controlled neuromodulation studies were summarized separately because they were not clinically comparable with usual-care, wait-list, or no-intervention control comparisons for pooled analysis. PMR was evaluated in only one small trial and was therefore summarized descriptively rather than pooled as an independent subgroup. Although the PMR study showed a large effect estimate, this finding should be interpreted cautiously because it was based on single-study evidence. For the three-arm trial with a shared comparator (Hum et al., 2019), sensitivity analyses using alternative handling of the shared control group did not materially change the overall interpretation of the findings. Sensitivity analyses were also conducted to examine the influence of endpoint versus change-score selection. Endpoint scores were used in the primary analysis whenever available. When change scores were available for the same study, the corresponding sensitivity analyses showed generally consistent directions of effect compared with the primary endpoint-score analysis. The direction and general magnitude of effects remained consistent with the primary endpoint-score analysis, suggesting that the main conclusions were not substantially influenced by the choice of endpoint versus change-score data. Therefore, the primary conclusions were considered robust to the choice of endpoint versus change-score data.

In studies reporting multiple validated depression scales, the prespecified primary scale was used in the main analysis to avoid double-counting participants. Alternative scales were examined in sensitivity analyses where possible, and the direction of effect remained generally consistent.

For the primary continuous outcome, subgroup analyses were conducted according to intervention category. CBT or cognitive-behavioral interventions, mindfulness-based interventions, and self-management or collaborative-care interventions were quantitatively pooled because each category was supported by at least two studies. Formal tests for subgroup differences were performed where sufficient data were available, but the results were interpreted cautiously because the number of studies within each subgroup was limited. Additional subgroup analyses and meta-regression according to age, epilepsy type or epileptogenic zone, seizure frequency, baseline depression status, follow-up duration and study quality were planned but not quantitatively performed because these characteristics were inconsistently reported across studies and the number of studies within each subgroup was limited. Follow-up duration varied across studies, ranging from immediate post-intervention assessment to long-term follow-up. According to the prespecified timepoint selection rule, the assessment closest to 3 months was selected for the primary analysis whenever available. Alternative follow-up assessments were considered in sensitivity analyses when extractable data were available. These factors were therefore considered descriptively when interpreting heterogeneity and subgroup findings. The main heterogeneity and sensitivity analyses are summarized in Table 8 and Supplementary Table S4.

### 3.7 Publication bias and small-study effects

For the primary continuous depression severity outcome, a funnel plot was generated to explore potential publication bias and small-study effects. Visual inspection did not suggest marked asymmetry, although several small studies showed relatively large effect estimates (Figure 6). Egger's regression test was performed using the 12 effect estimates included in the overall continuous depression severity analysis and did not indicate statistically significant funnel-plot asymmetry (intercept = −1.42, SE = 1.22, t = −1.17, *P* = 0.27). However, because the primary analysis included only 12 active-versus-control comparisons from 11 studies and the included interventions were clinically heterogeneous, the ability to detect publication bias was limited. Funnel plots and Egger's tests were not performed for intervention-category subgroup analyses because each subgroup contained fewer than ten studies. Therefore, publication bias and small-study effects could not be formally ruled out. For dichotomous outcomes, publication bias was not assessed because the number of studies was insufficient and outcome definitions varied substantially across studies.

**Figure 6:**
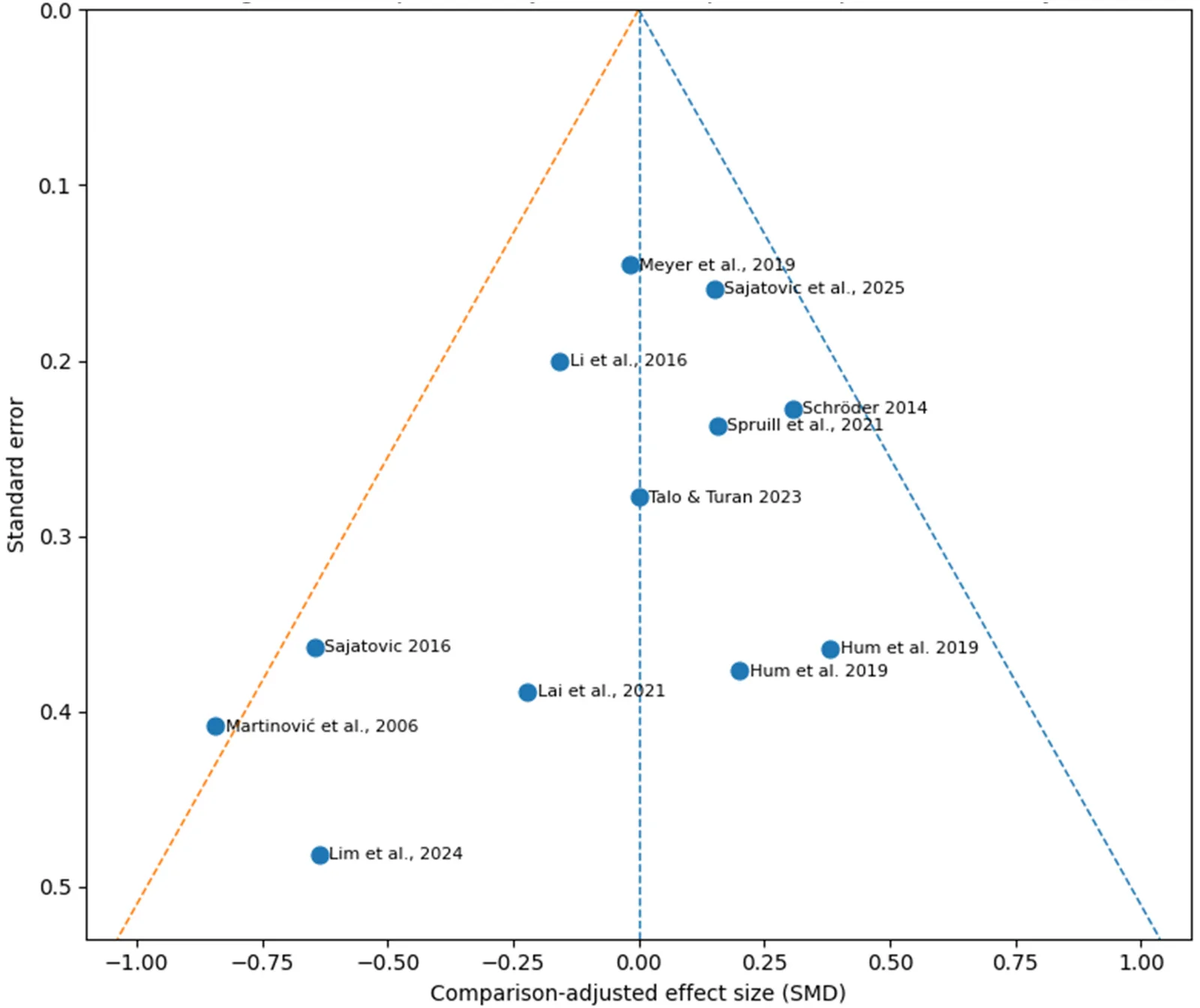
Funnel plot of active interventions versus TAU/control for continuous depression severity. The funnel plot shows comparison-level standardized mean differences against their standard errors for the primary continuous depression severity outcome. Negative SMD values favor active interventions over TAU/control. Visual inspection did not suggest marked asymmetry, but interpretation was limited by the small number of studies and clinical heterogeneity across intervention categories.

### 3.8 Summary of evidence and certainty assessment

#### 3.8.1 Summary of main findings

Overall, the evidence synthesis suggested that active psychological and behavioral interventions were associated with greater reductions in depression severity than TAU/control conditions. In the primary random-effects pairwise meta-analysis, the overall pooled estimate favored active interventions for continuous depression severity. Subgroup analyses further suggested beneficial effects of CBT or cognitive-behavioral interventions, mindfulness-based interventions, and self-management or collaborative-care interventions compared with TAU/control.

CBT or cognitive-behavioral interventions showed a statistically significant reduction in depression severity compared with TAU/control (SMD = −0.60, 95% CI: −1.07 to −0.12). Mindfulness-based interventions also showed a significant beneficial effect (SMD = −0.43, 95% CI: −0.76 to −0.11), with no observed statistical heterogeneity. Self-management or collaborative-care interventions were associated with a significant reduction in depression severity (SMD = −0.44, 95% CI: −0.79 to −0.10). PMR showed the largest study-level effect estimate (SMD = −1.67, 95% CI: −2.21 to −1.12), but this finding was based on a single small trial and was therefore summarized descriptively rather than pooled as an independent subgroup.

Taken together, CBT or cognitive-behavioral interventions and self-management or collaborative-care interventions showed the most consistent and clinically interpretable evidence for reducing depressive symptom severity in people with epilepsy. Mindfulness-based interventions may also be beneficial, although the certainty of evidence was lower. Although PMR showed a large effect in one trial, its robustness was limited by single-study evidence. Evidence for pharmacological head-to-head comparisons and neuromodulation-based interventions remained limited and was summarized separately.

#### 3.8.2 Certainty of evidence

The certainty of evidence for the primary outcome was assessed using the GRADE framework. The assessment considered risk of bias, inconsistency, indirectness, imprecision, and publication bias. For the primary continuous outcome of depression severity, the certainty of evidence was rated as low for CBT, mindfulness-based interventions, and self-management/care interventions, and very low for PMR (Table 10).

**Table 10:** Summary of findings for depression-related outcomes.

Outcome	Comparison	Studies (participants)	Effect estimate	Certainty
Depression severity	CBT vs. TAU/control	3 (308)	SMD = −0.55 (95% CI: −0.90 to −0.21)	⊕⊕◯◯ Low
Depression severity	MBCT/mindfulness vs. TAU/control	4 (151)	SMD = −0.46 (95% CI: −0.86 to −0.07)	⊕⊕◯◯ Low
Depression severity	Self-management/care vs. TAU/control	4 (334)	SMD = −0.43 (95% CI: −0.74 to −0.12)	⊕⊕◯◯ Low
Depression severity	PMR vs. TAU/control	1 (70)	SMD = −1.65 (95% CI: −2.32 to −0.98)	⊕◯◯◯ Very low
Adverse dichotomous depression outcomes	Pharmaceutical care vs. TAU	1 (143)	RR = 0.64 (95% CI: 0.42 to 0.99)	⊕⊕◯◯ Low
Response/remission outcome	Emyna + CAU vs. CAU	1 (200)	RR = 1.93 (95% CI: 1.11 to 3.38)	⊕⊕◯◯ Low

CBT, cognitive behavioral therapy; MBCT, mindfulness-based cognitive therapy; PMR, progressive muscle relaxation; TAU, treatment as usual; CAU, care as usual; SMD, standardized mean difference; RR, risk ratio; CI, confidence interval. Negative SMD values favor the intervention for continuous depression severity outcomes. For adverse dichotomous depression outcomes, RR values <1 favor the intervention; for response/remission outcomes, RR values >1 favor the intervention. Certainty of evidence was assessed using the GRADE framework.

Although PMR showed the largest study-level effect estimate, the certainty of evidence was rated as very low because this comparison was supported by only one small trial and could not be quantitatively pooled as an independent subgroup. In addition, the potential influence of small-study effects and publication bias could not be formally excluded. Therefore, the PMR finding should be interpreted cautiously. Detailed certainty assessments are provided in Supplementary Table S2.

For selected secondary dichotomous outcomes, pharmaceutical care reduced clinically significant depressive symptoms compared with TAU, and Emyna plus care as usual improved clinically significant response compared with care as usual alone. However, both estimates were supported by single-study evidence and were therefore interpreted cautiously. The certainty of evidence for these dichotomous outcomes was rated as low or very low, depending on the degree of imprecision and risk-of-bias concerns.

## 4 Discussion

### 4.1 Principal findings

This systematic review and meta-analysis evaluated the efficacy of psychological and behavioral interventions for depressive symptoms in people with epilepsy, with supplementary evidence from pharmacological and neuromodulation-based studies when depression-related outcomes were reported. A total of 22 studies were included in the systematic review. For the primary outcome of depression severity measured using validated rating scales, 11 studies contributed 12 active-versus-control comparisons to the quantitative synthesis. The primary random-effects pairwise meta-analysis suggested that active psychological and behavioral interventions were associated with greater reductions in depression severity compared with TAU/control.

In subgroup analyses, CBT or cognitive-behavioral interventions, mindfulness-based interventions, and self-management or collaborative-care interventions all showed significant beneficial effects compared with TAU/control. CBT showed a moderate reduction in depression severity, while mindfulness-based interventions and self-management or collaborative-care interventions showed smaller but statistically significant effects. PMR showed the largest study-level effect estimate, but this finding was based on a single small trial and was therefore summarized descriptively rather than pooled as an independent subgroup. Thus, PMR should be considered a promising but still uncertain intervention rather than a definitively superior option.

For dichotomous depression-related outcomes, the available evidence was more limited and heterogeneous. Pharmaceutical care significantly reduced the proportion of patients with clinically significant depressive symptoms compared with TAU. UPLIFT and cognitive-behavioral intervention also showed favorable point estimates for adverse depression outcomes, although their confidence intervals were wide and crossed the null value. For response or remission outcomes, Emyna plus care as usual was associated with a higher probability of clinically significant improvement compared with care as usual alone, while PEARLS showed a favorable but imprecise effect estimate. Active head-to-head comparisons, including escitalopram versus venlafaxine and sertraline versus CBT, did not show clear differences in response or remission. Because dichotomous outcomes differed in definition, comparator type, and direction of benefit, they were summarized descriptively rather than pooled into a single overall estimate.

### 4.2 Comparison with previous reviews

The findings of the present review are broadly consistent with previous systematic reviews suggesting beneficial effects of CBT and mindfulness-based interventions on depressive symptoms and psychological well-being in people with epilepsy (35–37). However, the present review extends the existing evidence in several important respects. First, previous reviews have generally focused on individual intervention categories, particularly CBT or mindfulness-based approaches, whereas we synthesized evidence across multiple clinically relevant psychological and behavioral intervention categories within the same analytical framework. This allowed the evidence for CBT, mindfulness-based interventions, self-management or collaborative-care approaches, and relaxation-based interventions to be evaluated using consistent eligibility criteria, outcome definitions, and analytical methods. Second, rather than combining depression with broader psychosocial outcomes such as anxiety, quality of life, or general psychological well-being, the present review focused specifically on depression-related outcomes. Third, we separately evaluated continuous depression severity and clinically relevant dichotomous depression-related outcomes, including clinically significant depressive symptoms, depression incidence, treatment response, and remission, thereby preserving differences in their clinical interpretation and direction of benefit. Finally, the present review incorporated intervention-category subgroup analyses and GRADE assessment, allowing treatment effects to be interpreted alongside the consistency and certainty of the available evidence. Taken together, these features provide a more depression-focused and clinically structured synthesis of psychological and behavioral interventions for people with epilepsy than previous reviews.

### 4.3 Interpretation of the primary outcome

In addition to reducing depressive symptoms, psychological and behavioral interventions may provide broader benefits by improving coping skills, illness understanding, self-efficacy, behavioral activation, and social support.

CBT-related interventions showed a statistically significant benefit compared with TAU/control. This finding is consistent with previous systematic reviews suggesting that CBT may improve depressive symptoms and quality of life in people with epilepsy, although effects on seizure control remain less certain (38, 39). This result is clinically plausible because CBT directly targets maladaptive cognitions, avoidance behaviors, negative illness perceptions, and emotional responses associated with chronic neurological disease. Internet-based CBT programs, such as Deprexis and Emyna, may be particularly useful for people with epilepsy because access to face-to-face psychotherapy may be limited by driving restrictions, seizure unpredictability, stigma, and lack of specialized mental health services.

Mindfulness-based interventions also showed beneficial effects. This is broadly consistent with previous evidence indicating that mindfulness-based interventions may improve psychological well-being in people with epilepsy, although the overall evidence remains limited and further trials are still needed (36). Mindfulness-based approaches may reduce depressive symptoms by improving emotional regulation, increasing awareness of negative thought patterns, and reducing stress reactivity. For people with epilepsy, these interventions may also help patients manage seizure-related uncertainty, anticipatory anxiety, perceived loss of control, and psychosocial stress. However, the magnitude of benefit was modest, and included studies varied in intervention intensity, duration, delivery format, and outcome measurement.

Self-management and collaborative-care interventions were associated with reduced depression severity. These findings are clinically plausible because self-management programs may improve illness understanding, medication adherence, coping strategies, access to psychosocial support, and confidence in managing epilepsy-related challenges (40, 41). These interventions are particularly attractive in routine epilepsy care because they can be embedded into multidisciplinary clinical pathways and delivered by nurses, pharmacists, trained care managers, or other healthcare professionals. Although the pooled effect size was moderate, the evidence may be more generalizable than single-study interventions because this category included several pragmatic care models.

### 4.4 Interpretation of dichotomous outcomes

Dichotomous outcomes provided complementary clinical information but were less suitable for formal pooled analysis. The included studies reported different binary outcomes, including depression incidence, clinically significant depressive symptoms, moderate-to-severe depression, treatment response, clinically significant improvement, and remission. These outcomes differ in clinical meaning and direction of benefit; therefore, adverse depression outcomes and response/remission outcomes were summarized separately.

For adverse depression outcomes, lower event rates favor the intervention. Pharmaceutical care showed a statistically significant reduction in clinically significant depressive symptoms, suggesting that structured medication review, adherence support, patient education, and seizure monitoring may improve mental health outcomes in people with epilepsy. UPLIFT and cognitive-behavioral intervention also showed favorable point estimates, but the uncertainty was substantial because of small sample sizes, low event numbers, and wide confidence intervals.

For response/remission outcomes, higher event rates favor the intervention. Emyna plus CAU significantly improved clinically meaningful response compared with CAU alone, while PEARLS showed a favorable but imprecise effect. These findings suggest that structured psychological and collaborative-care programs may increase the likelihood of clinically meaningful improvement. However, because most binary estimates were supported by single studies, the certainty of these findings remains limited.

### 4.5 Certainty of evidence

The certainty of evidence varied across intervention categories. Using the GRADE framework, the evidence for the primary outcome of depression severity was rated as low for CBT, mindfulness-based interventions, and self-management/care interventions, and very low for PMR. The main reasons for downgrading included some concerns regarding risk of bias, imprecision, sparse evidence, heterogeneity, and limited ability to assess publication bias (42).

The very-low certainty for PMR is particularly important. Although PMR showed the largest study-level effect estimate, this result was based on a single small trial and was not quantitatively pooled as an independent subgroup. Therefore, the apparent large effect should not be overinterpreted. CBT, mindfulness-based interventions, and self-management/care interventions showed more moderate effect estimates, but the certainty of evidence remained low because of methodological limitations, small numbers of studies, and heterogeneity across trials. For dichotomous outcomes, the certainty of evidence was generally low because few studies contributed data, event numbers were small, and outcome definitions differed across trials. Therefore, binary outcome findings should be considered supportive rather than confirmatory.

### 4.6 Risk of bias and publication bias

Most included studies were judged as having some concerns according to the Cochrane RoB 2 tool. The main sources of bias were related to the difficulty of blinding participants and personnel in psychological or behavioral interventions, frequent use of self-reported depression scales, incomplete outcome data in some studies, and limited reporting of prespecified outcomes. One study was judged as having high risk of bias, mainly because of missing outcome data in long-term follow-up.

For publication bias, a funnel plot was generated for the primary continuous depression severity outcome. Visual inspection did not suggest marked asymmetry, although several small studies showed relatively large effect estimates. Because only 12 active-versus-control comparisons from 11 studies contributed to the primary continuous outcome analysis, and because included interventions were clinically heterogeneous, the ability to detect publication bias or small-study effects was limited. Therefore, publication bias could not be formally ruled out. For dichotomous outcomes, publication bias was not formally assessed because the number of studies was insufficient and outcome definitions varied substantially.

### 4.7 Strengths of this study

This review has several strengths. First, it systematically evaluated a broad range of interventions for depressive symptoms in people with epilepsy, including psychological therapy, mindfulness-based approaches, self-management programs, collaborative care, pharmaceutical care, pharmacological treatment, and neuromodulation. Second, continuous depression severity and dichotomous depression-related outcomes were analyzed separately, avoiding inappropriate pooling of outcomes with different effect measures and clinical directions. Third, SMD was used for continuous outcomes to account for the use of different depression rating scales across studies. Fourth, subgroup analyses by intervention category were conducted for clinically comparable active-versus-control comparisons. Fifth, risk of bias and certainty of evidence were systematically assessed, allowing the findings to be interpreted not only according to effect size but also according to evidence quality.

### 4.8 Clinical interpretation and durability of treatment effects

The magnitude of pooled effects should be interpreted in relation to clinically meaningful differences in depressive symptom severity. Because depression outcomes were measured using different instruments across studies, direct conversion of SMDs into a specific scale score was not consistently possible. According to conventional thresholds, SMDs of approximately 0.2, 0.5, and 0.8 are generally considered small, moderate, and large effects, respectively. Therefore, the pooled effects observed for CBT (SMD = −0.55) and mindfulness-based interventions (SMD = −0.46) indicate moderate reductions in depressive symptoms, whereas the PMR estimate (SMD = −1.65) represents a large effect. However, this estimate was derived from only one small trial and should be interpreted cautiously.

### 4.9 Durability and sustainability of intervention effects

The durability of treatment effects is clinically important but remains insufficiently established in the current evidence base. Follow-up duration varied substantially across the included studies, ranging from short-term assessment at 5 days to longer-term follow-up of up to 18 months. However, follow-up schedules were not standardized across trials, and many studies primarily reported outcomes at the end of treatment or at relatively short-term follow-up. Consequently, the available evidence does not allow reliable quantitative comparisons of the persistence of treatment effects across different intervention categories.

For CBT, mindfulness-based interventions, and self-management or collaborative-care approaches, favorable effects were observed at the follow-up time points reported in individual studies, but evidence regarding maintenance of these benefits after completion of the active intervention remained limited and inconsistent. This issue is particularly relevant for psychological and behavioral interventions because treatment effects may diminish over time when structured therapeutic contact or behavioral reinforcement is discontinued. For PMR, conclusions regarding durability are even more uncertain because the evidence was derived from a single small trial.

Future trials should incorporate standardized long-term follow-up assessments, for example at 3, 6, and 12 months after completion of treatment, to determine whether initial improvements are sustained. Strategies aimed at maintaining treatment benefits should also be evaluated, including booster sessions, continued self-management support, digitally delivered reinforcement or monitoring, and integration of psychological and behavioral interventions into routine epilepsy care. Such approaches may help transform short-term improvements in depressive symptoms into more durable clinical benefits.

### 4.10 Novel findings and added value

A notable finding of the present review was that the magnitude and consistency of treatment effects differed across intervention categories, indicating that psychological and behavioral interventions should not be regarded as a single homogeneous treatment class. CBT and self-management/collaborative-care interventions showed relatively consistent beneficial effects across the available trials, while mindfulness-based interventions also demonstrated benefit but with variation in intervention format, intensity, and outcome assessment. Importantly, the certainty of evidence for these three intervention categories remained low, indicating that the observed benefits should be interpreted cautiously despite generally favorable effect estimates.

Another important finding was the contrast between effect magnitude and evidence certainty. Progressive muscle relaxation showed the largest observed effect estimate, but this finding was based on a single small trial and was supported by very-low-certainty evidence. Therefore, a large effect size should not be interpreted as evidence of superior effectiveness when the underlying evidence base is sparse. The present review also highlights the value of considering both effect magnitude and certainty of evidence when interpreting psychological and behavioral interventions for depressive symptoms in people with epilepsy.

## 5 Limitations

Several limitations should be acknowledged. First, the number of available studies was limited, and several intervention categories were supported by only a small number of trials. PMR was supported by only one small study, so its large effect estimate should be considered hypothesis-generating rather than definitive.

Second, some intervention categories were clinically heterogeneous. For example, CBT included both internet-based CBT and cognitive-behavioral intervention, whereas self-management/care included multidisciplinary management, collaborative care, pharmaceutical care, education, and solution-focused therapy. Although grouping was necessary to allow quantitative synthesis, it may have introduced clinical heterogeneity.

Third, depression outcomes were measured using different scales, including BDI, HAM-D, MADRS, PHQ-9, QIDS, NDDI-E, and CES-D. Although SMD was used to standardize effects, differences in scale properties, cutoffs, assessment timing, and clinical interpretation may still affect comparability. In addition, follow-up durations varied substantially across studies, ranging from immediate post-intervention assessments to longer-term follow-up. Although a prespecified timepoint selection strategy was applied, differences in follow-up duration may have influenced the magnitude and sustainability of intervention effects. Future trials with standardized follow-up schedules are needed to better evaluate the durability of treatment effects.

Fourth, dichotomous outcomes were inconsistently defined across studies. Depression incidence, clinically significant depressive symptoms, treatment response, and remission are not interchangeable outcomes and were therefore summarized separately. This reduced the number of studies available for each binary outcome category and limited the possibility of formal pooling.

Fifth, several potential effect modifiers may have contributed to heterogeneity across studies. These included baseline depressive symptom severity, delivery modality, intervention dose and duration, outcome scale type, follow-up duration, age group, epilepsy type, epileptogenic zone, seizure frequency, antiseizure medication burden, and study quality. For example, internet-based interventions may differ from in-person psychological interventions in treatment engagement, adherence, and therapist contact, while short-term post-treatment effects may differ from longer-term maintenance effects. Similarly, epilepsy-specific depression measures, such as NDDI-E, may not be directly comparable with broader depression scales, such as PHQ-9, BDI, CES-D, or HAM-D. Because these variables were inconsistently reported and the number of studies within each subgroup was limited, formal meta-regression or additional quantitative subgroup analyses were not feasible. Therefore, these potential moderators were considered descriptively, and future trials should report these variables more consistently. Sixth, some estimates were based on reconstructed or converted data, such as conversion from standard error to standard deviation or reconstruction from reported percentages. Although standard methods were used, these data-processing steps may have introduced additional uncertainty.

Finally, most studies had some concerns regarding risk of bias, and publication bias could not be formally excluded because of the limited number of studies. These limitations suggest that the findings should be interpreted cautiously, particularly for intervention categories supported by sparse or single-study evidence.

## 6 Clinical implications

The findings suggest that non-pharmacological interventions should be considered as important components of depression management in people with epilepsy, consistent with existing literature emphasizing the need to address psychiatric comorbidities alongside seizure control (43–45). CBT, mindfulness-based interventions, and self-management/care programs may provide clinically meaningful reductions in depressive symptoms, with relatively favorable feasibility and safety profiles. These approaches may be particularly valuable for patients who have concerns about antidepressant use, drug interactions, adverse effects, or seizure-related medication issues.

In routine epilepsy care, multidisciplinary and collaborative-care models may be especially practical because they can integrate psychological screening, medication adherence support, seizure education, and referral pathways. Internet-based or telephone-delivered programs may improve access for patients with mobility restrictions, driving limitations, stigma, or limited availability of mental health specialists. PMR may be a promising low-cost intervention, but current evidence is insufficient to recommend it as the most reliable strategy. Larger randomized trials are needed to clarify its effect.

## 7 Implications for future research

Future trials should use adequately powered randomized designs, standardized depression outcomes, and longer follow-up. Studies should report both continuous depression scores and clinically meaningful dichotomous outcomes, such as response, remission, clinically significant depression, and depression incidence. Greater consistency in outcome definitions would improve the comparability of future meta-analyses.

Future research should also improve reporting of epilepsy-specific characteristics, including epilepsy type, seizure frequency, epileptogenic zone, antiseizure medication burden, baseline depression severity, and psychiatric comorbidity. These data would allow more informative subgroup and meta-regression analyses. In addition, future studies should include standardized reporting of adverse events, treatment adherence, intervention fidelity, patient preference, and implementation feasibility.

Head-to-head trials comparing active interventions, such as CBT versus mindfulness-based interventions, CBT versus self-management/care, and psychological therapy versus pharmacological treatment, are also needed. Such trials would help clarify which interventions are most appropriate for different patient groups and clinical contexts.

## 8 Conclusions

In this systematic review and meta-analysis, psychological, mindfulness-based, and self-management/care interventions were associated with reductions in depression severity among people with epilepsy compared with TAU/control. CBT and self-management/care interventions showed moderate-certainty evidence and may represent more reliable and clinically applicable strategies for improving depressive symptoms in this population. MBCT/mindfulness-based interventions also showed beneficial effects, although the certainty of evidence was lower. PMR showed a large effect in one small trial, but the evidence was very-low certainty and should be interpreted cautiously. Further high-quality, adequately powered randomized trials are needed to confirm the effectiveness of these interventions and to clarify which approaches are most suitable for different groups of people with epilepsy.

## Data Availability

The original contributions presented in the study are included in the article/Supplementary Material, further inquiries can be directed to the corresponding author.
