# Supplementary material for: Efficacy of psychological and behavioral interventions for depressive symptoms in people with epilepsy: a systematic review and meta-analysis
Source: Front Med (Lausanne). 2026 Sep 17;13:1917962. doi: 10.3389/fmed.2026.1917962 (PMC13627414; doi:10.3389/fmed.2026.1917962)
Supplement: Supplementary file 1 [file Supplementaryfile1.pdf]

Supplementary Material

Supplementary Table S1. Database-specific search strategies

Database	Search date	Search strategy
PubMed	May 31, 2026	*(Epilepsy[Mesh] OR epilepsy OR epileptic OR seizure OR convulsion*) AND (Depression[Mesh] OR depress* OR depressive symptom* OR depressive disorder* OR mood disorder* OR psychological distress) AND (intervention* OR therapy OR treatment OR psychotherapy OR cognitive behavioral therapy OR CBT OR mindfulness OR self-management OR collaborative care OR relaxation OR pharmaceutical care OR pharmacological intervention OR neuromodulation OR transcranial direct current stimulation OR tDCS OR rTMS) AND (randomized controlled trial[Publication Type] OR controlled clinical trial[Publication Type] OR randomized OR randomised OR trial OR intervention study)**
MEDLINE (via PubMed/Ovid as applicable)	May 31, 2026	*(exp Epilepsy/ OR epilepsy OR epileptic OR seizure OR convulsion*) AND (exp Depression/ OR depress* OR depressive symptom* OR depressive disorder* OR mood disorder*) AND (therapy OR intervention* OR treatment OR cognitive behavioral therapy OR CBT OR mindfulness OR self-management OR collaborative care OR relaxation OR pharmaceutical care OR pharmacological treatment OR neuromodulation OR tDCS OR rTMS) AND (randomized controlled trial OR controlled clinical trial OR random* OR trial OR intervention study)**
Embase	May 31, 2026	*('epilepsy'/exp OR epilepsy OR epileptic OR seizure OR convulsion*) AND ('depression'/exp OR depress* OR depressive symptom* OR depressive disorder* OR mood disorder*) AND ('psychotherapy'/exp OR intervention* OR treatment OR therapy OR 'cognitive behavioral therapy' OR CBT OR mindfulness OR self-management OR collaborative care OR relaxation OR pharmaceutical care OR pharmacological intervention OR neuromodulation OR 'transcranial direct current stimulation'

Database	Search date	Search strategy
		OR tDCS OR rTMS) AND ('randomized controlled trial'/exp OR random* OR trial OR controlled study OR intervention study)**
Web of Science Core Collection	May 31, 2026	*TS=(epilepsy OR epileptic OR seizure OR convulsion*) AND TS=(depress* OR depressive symptom* OR depressive disorder* OR mood disorder*) AND TS=(intervention* OR therapy OR treatment OR psychotherapy OR "cognitive behavioral therapy" OR CBT OR mindfulness OR self-management OR "collaborative care" OR relaxation OR pharmaceutical care OR pharmacological intervention OR neuromodulation OR tDCS OR rTMS) AND TS=(random* OR trial OR controlled OR intervention study)**
Cochrane Library	May 31, 2026	*([Epilepsy] OR epilepsy OR seizure):ti,ab,kw AND ([Depression] OR depress* OR depressive symptom* OR mood disorder*):ti,ab,kw AND (intervention* OR therapy OR treatment OR psychotherapy OR CBT OR mindfulness OR self-management OR collaborative care OR relaxation OR pharmaceutical care OR pharmacological intervention OR neuromodulation):ti,ab,kw**

Supplementary Table S2 GRADE summary of findings for continuous depression severity

Comparison	No. of studies	Effect estimate	Risk of bias	Inconsistency	Indirectness	Imprecision	Publication bias	Certainty
CBT vs TAU/control	3	SMD -0.60 (-0.90 to -0.21)	Serious	Serious	Not serious	Not serious	Not assessable; no downgrade	Low

Comparison	No. of studies	Effect estimate	Risk of bias	Inconsistency	Indirectness	Imprecision	Publication bias	Certainty
MBCT/mindfulness vs TAU/control	4	SMD -0.46 (-0.86 to -0.07)	Serious	Not serious	Not serious	Serious	Not assessable; no downgrade	Low
Self-management/care vs TAU/control	4	SMD -0.43 (-0.74 to -0.12)	Serious	Serious	Not serious	Not serious	Not assessable; no downgrade	Low
PMR vs TAU/control	1	SMD -1.65 (-2.32 to -0.98)	Serious	Not estimable	Not serious	Serious	Not estimable; single small positive trial	Very low

Note: Certainty of evidence was assessed using the GRADE framework. Evidence was downgraded for risk of bias when most contributing studies had some concerns or high risk of bias according to RoB 2 assessment. Inconsistency was judged according to heterogeneity estimates and overlap of confidence intervals. Imprecision was judged according to sample size, confidence interval width, and whether estimates were based on sparse or single-study evidence. Publication bias was judged as undetected when funnel-plot assessment did not suggest clear asymmetry, but it was considered not estimable for single-study comparisons.

Supplementary Table S3. Detailed intervention characteristics, depression assessment instruments, analyzed follow-up timepoints, attrition, and role in evidence synthesis of included studies

Study	Sample size, randomized; analyzed	Intervention category and delivery modality	Intervention dose/duration	Comparator	Depression instrument	Baseline depression status/severity	Primary timepoint analyzed	Attrition	Role in evidence synthesis
Schröder 2014	38/40; analysis is based on 38/40	CBT; internet-based Deprexis program	9-week online intervention	Wait-list control	BDI-I	Participants had depressive symptoms; exact arm-level baseline mean NR	9 weeks	Exact attrition NR	Primary continuous-outcome analysis
Talo & Turan 2023	35/35; 35/35 analyzed	Relaxation-based; instructed PMR exercises	30 min/day, 3 days/week for 4 weeks	No intervention	BDI	BDI >9 required; IG 28.31±13.13, CG 28.40±11.57	4 weeks	No attrition evident from analyzed sample	Primary continuous-outcome analysis; single-study PMR evidence
Sajatovic et al. 2016	22/22 randomized; 19/16 analyzed	Self-management/psychoeducational; group-based TIME with	12 group sessions plus 4 telephone mainten	TAU	MADRS; PHQ-9 in sensitivity	MADRS: 16.75±10.28 vs 22.94±11.81; PHQ-9:	16 weeks	3/22 vs 6/22 lost; 19/16	Primary continuous outcome; alternati

Study	Sample size, randomized; analyzed	Intervention category and delivery modality	Intervention dose/duration	Comparator	Depression instrument	Baseline depression status/severity	Primary timepoint analyzed	Attrition	Role in evidence synthesis
		telephone maintenance	ance sessions; 16 weeks		analysis	9.70±5.55 vs 11.76±5.72		completed	ve-scale sensitivity analysis
Lai et al. 2021	14/14 randomized; 14/14 at 6 weeks; approximately 11/14 in the intervention arm at 12 weeks	Mindfulness-based; structured mindfulness training	6-week intervention; additional 12-week follow-up	Wait-list/TAU	BDI-II	Adults with epilepsy; exact baseline BDI-II means NR	6 weeks	Incomplete 12-week follow-up; intervention arm 11/14	Primary continuous outcome; 12-week sensitivity evidence
Lim et al. 2024	10/10 randomized; approximately 9/10 in the	Mindfulness-based; video-guided mindful breathing	20-minute video-guided training	Wait-list control	NDDI-E	Elevated depressive or anxiety symptoms; exact	2 weeks	One intervention participant unav	Primary continuous outcome; 6-week sensitivi

Study	Sample size, randomized; analyzed	Intervention category and delivery modality	Intervention dose/duration	Comparator	Depression instrument	Baseline depression status/severity	Primary timepoint analyzed	Attrition	Role in evidence synthesis
	intervention arm at 6 weeks					baseline means NR		ailable at 6-week assessment	ty evidence
Spruill et al. 2021	36/36	Mindfulness/self-management; Project UPLIFT, remotely delivered	Intervention schedule NR; assessments at 6 and 12 months	TAU/epilepsy education	PHQ-9	Elevated depressive symptoms; exact baseline PHQ-9 means NR	6 months	Longer-term attrition reported, but exact arm-level counts NR	Primary continuous outcome; dichotomous and follow-up sensitivity evidence
Meyer et al. 2019	100/100	CBT; internet-based Emyna plus CAU	Internet program over approximately 3 months	CAU	PHQ-9	Exact baseline PHQ-9 means NR	3 months	Incomplete follow-up noted; exact	Primary continuous outcome and clinically significa

Study	Sample size, randomized; analyzed	Intervention category and delivery modality	Intervention dose/duration	Comparator	Depression instrument	Baseline depression status/severity	Primary timepoint analyzed	Attrition	Role in evidence synthesis
								arm-level counts NR	nt improvement analysis
Sajatovic et al. 2025	80/80	Self-management/psychosocial; SMART intervention	6-month intervention/follow-up period; session dose NR	Wait-list control	PHQ-9	Exact baseline PHQ-9 means NR	6 months	Exact attrition NR	Primary continuous-outcome analysis
Li et al. 2016	52/52	Self-management/psychosocial; short-term family therapy plus AEDs	Treatment period 3 months; session frequency NR	AEDs alone	HAM-D	Exact baseline HAM-D means NR	3 months	Exact attrition NR	Primary continuous-outcome analysis
Martinović et al. 2006	15/15	CBT/cognitive-behavioral intervention; delivery format NR	Intervention and follow-up period 9 months; session dose NR	TAU/supportive counselling	HAM-D; BDI and CES-D in sensitivity analyses	Adolescents with epilepsy; exact baseline means NR	9 months	Exact attrition NR	Primary continuous outcome and adverse dichotomous outcome

Study	Sample size, randomized; analyzed	Intervention category and delivery modality	Intervention dose/duration	Comparator	Depression instrument	Baseline depression status/severity	Primary timepoint analyzed	Attrition	Role in evidence synthesis
Gilliam et al. 2019	72/68	Active head-to-head comparison; sertraline versus CBT	16 weeks; drug dose and CBT session frequency NR in the extraction table	CBT as active comparator	BDI; CES-D sensitivity measure; MINI remission	Participants had epilepsy and depression; exact baseline means NR	16 weeks	Exact arm-level attrition NR	Separate active head-to-head continuous and remission analyses
Losada-Camacho 2022	70/74 randomized; 69/74 analyzed	Pharmaceutical care; pharmacist-led care program	6 months	TAU	CES-D	CES-D: 20.75±11.64 vs 19.65±12.05; CES-D ≥20: 34/69 vs 36/74	6 months	One intervention participant not analyzed; 69/74 completed	Adverse dichotomous depression-outcome analysis
Zheng 2019	92/92	Multidisciplinary management; multidisciplinary clinical-care program	12 months	Routine AED treatment/education	Depression outcome mea	Moderate-to-severe depression outcom	12 months	Exact attrition NR	Adverse dichotomous depression-outcom

Study	Sample size, randomized; analyzed	Intervention category and delivery modality	Intervention dose/duration	Comparator	Depression instrument	Baseline depression status/severity	Primary timepoint analyzed	Attrition	Role in evidence synthesis
					sure not clearly specified	e reported; exact baseline severity NR			e analysis
Thompson 2014	62/56 randomized; MDD event analysis 52/56	Mindfulness-based CBT/self-management; Project UPLIFT via web/telephone	10-week program	TAU/wait-list	mBDI, BDI and PHQ-9; MDD incidence	mBDI 20.3 vs 20.2; BDI 7.3 vs 7.2; PHQ-9 6.7 vs 5.8	10 weeks	MDD analysis included 52/56; 10 intervention participants lacked event data	Adverse dichotomous depression-outcome analysis
Ciechanowski 2010	40/40 randomized; response analys	Collaborative care; PEARLS depression-care intervention	12-month collaborative-care program	TAU	HSCL-20	HSCL-20: 2.1±0.6 vs 1.9±0.6	12 months	Response data available for	Response-outcome analysis

Study	Sample size, randomized; analyzed	Intervention category and delivery modality	Intervention dose/duration	Comparator	Depression instrument	Baseline depression status/severity	Primary timepoint analyzed	Attrition	Role in evidence synthesis
	is 35/29							35/29; 5/11 participants without response data	
Chaytor 2011	Original cohort 40/40; 18-month analysis is 30/28	Collaborative-care follow-up; PEARLS long-term assessment	Follow-up to 18 months	TAU	HSCL-20	Same/overlapping PEARLS cohort; baseline approximately 2.1±0.6 vs 1.9±0.6	18 months	10/40 vs 12/40 not represented in 18-month response analysis	Sensitivity evidence only because of probable overlap with Ciechanowski 2010
Bhatele et al. 2023	45/45 randomized; 42/40 per-	Pharmacological active comparison	Escitalopram 5–20 mg/day versus venlafaxi	Venlafaxine	HAM-D	13.53±3.27 vs 13.02±3.57; mild-to-	8 weeks	3/45 vs 5/45 excluded	Separate active head-to-head continu

Study	Sample size, randomized; analyzed	Intervention category and delivery modality	Intervention dose/duration	Comparator	Depression instrument	Baseline depression status/severity	Primary timepoint analyzed	Attrition	Role in evidence synthesis
	protocol		ne 37.5–150 mg/day for 8 weeks			moderate depression		from per-protocol analysis	ous, response and remission analyses
Mota 2021	13/13 randomized; 11/12 completed	Neuromodulation; home-based tDCS plus TAU	Protocol duration approximately 60 days; stimulation dose NR in current extraction	Sham tDCS plus TAU	BDI-II	Adults with temporal lobe epilepsy and depressive symptoms; exact baseline means NR	60 days	2/13 vs 1/13 did not complete	Separate sham-controlled neuromodulation evidence
Li et al. 2025	53/63 randomized; 46/49 completed at 12	Antiseizure-medication head-to-head comparison	Lacosamide monotherapy versus oxcarbazepine monothe	Oxcarbazepine monotherapy	HAM-D	Adolescents with focal epilepsy and depression;	12 months	7/53 vs 14/63 did not complete the	Separate AED head-to-head comparison

Study	Sample size, randomized; analyzed	Intervention category and delivery modality	Intervention dose/duration	Comparator	Depression instrument	Baseline depression status/severity	Primary timepoint analyzed	Attrition	Role in evidence synthesis
	months		rapy; 6- and 12-month assessments			exact baseline means NR		12-month assessment	
Zhang & Zhao 2020	53/55	Pharmacological/AED-combination head-to-head comparison	Oxcarbazepine plus escitalopram versus lamotrigine plus escitalopram for 6 months	Lamotrigine plus escitalopram	HAM-D; MADRS sensitivity measure	HAM-D 21.11±1.37 vs 21.13±1.35; MADRS 29.33±2.21 vs 29.22±2.25	6 months	Exact attrition NR	Separate active head-to-head comparison
Liu et al. 2016	21/12	Neuromodulation; active tDCS	Short-term tDCS protocol; assessments at 5 days, 2 weeks and 4 weeks	Sham stimulation	BDI and NDDI-E change scores	Temporal lobe epilepsy; exact baseline scores NR	5 days/nearest post-treatment assessment	Exact attrition NR	Separate sham-controlled neuromodulation evidence

Study	Sample size, randomized; analyzed	Intervention category and delivery modality	Intervention dose/duration	Comparator	Depression instrument	Baseline depression status/severity	Primary timepoint analyzed	Attrition	Role in evidence synthesis
Hum et al. 2019	UPLIFT 20/EpINFO 24/WLC 11; per-protocol arm sizes	Three-arm remotely delivered intervention: UPLIFT mindfulness-based CBT and EpINFO self-management/education	8-week distance-delivered programs; follow-up at 6 months and 1 year	Wait-list control	QIDS primary; NDDI-E sensitivity measure	Self-reported depressive symptoms; CESD-R ≥12 required	8 weeks	Per-protocol analysis; additional arm-level attrition beyond stated sample sizes NR	Two primary continuous comparisons with shared-control adjustment

Abbreviations: AED, antiseizure/antiepileptic drug; BDI, Beck Depression Inventory; CAU, care as usual; CES-D, Center for Epidemiologic Studies Depression Scale; CBT, cognitive behavioral therapy; HAM-D, Hamilton Depression Rating Scale; HSCL-20, Hopkins Symptom Checklist-20; IG, intervention group; MADRS, Montgomery–Åsberg Depression Rating Scale; NDDI-E, Neurological Disorders Depression Inventory for Epilepsy; NR, not reported or not available in the extracted data; PHQ-9, Patient Health Questionnaire-9; PMR, progressive muscle relaxation; QIDS, Quick Inventory of Depressive Symptomatology; TAU, treatment as usual; tDCS, transcranial direct current stimulation; UPLIFT, Using Practice and Learning to Increase Favorable Thoughts; WLC, wait-list control. Sample sizes are presented as randomized arm sizes followed by analyzed/completer samples when these differed. For the three-arm Hum et al. trial, the

shared wait-list comparator was divided equally across the two pairwise comparisons in the overall meta-analysis.

Supplementary Table S4. Summary of sensitivity analyses in the pairwise meta-analysis

Sensitivity analysis	Main purpose	Result	Robustness
Main random-effects pairwise meta-analysis	Primary analysis of continuous depression severity	CBT, MBCT/mindfulness-based interventions, self-management/care interventions, and PMR showed greater reductions in depressive symptoms than TAU/control	Reference analysis
Excluding high-risk-of-bias study	Assess the influence of studies at high risk of bias	No material change was observed in the direction or interpretation of the main findings	Robust
Excluding Chaytor 2011 from binary analysis	Avoid potential overlap with the Ciechanowski 2010 PEARLS trial	The main conclusions for dichotomous outcomes were not materially altered	Robust
Excluding non-comparable comparisons	Assess the influence of pharmacological head-to-head and sham-controlled neuromodulation comparisons	These comparisons were summarized separately and did not affect the primary pooled estimates	Robust
Treating PMR as single-study evidence	Assess the influence of a comparison supported by only one small trial	PMR showed a large effect, but the estimate was based on one small trial and was therefore interpreted cautiously	Cautious interpretation required

Sensitivity analysis	Main purpose	Result	Robustness
Using alternative depression scales	Assess the influence of scale selection in studies reporting multiple depression measures	The direction of effect remained generally consistent across alternative depression scales	Robust
Endpoint-score primary analysis	Use the most consistently reported continuous depression outcome	Endpoint scores were used in the primary analysis whenever available	Reference analysis
Using change scores where available	Assess the influence of endpoint versus change-score selection	The direction of effect remained generally consistent, and the main conclusions were not materially altered	Robust
Using alternative depression scales	Assess the influence of scale selection in studies reporting multiple depression measures	The direction of effect remained generally consistent across alternative depression scales	Robust
